# ^15^N-Labelling and structure determination of adamantylated azolo-azines in solution

**DOI:** 10.3762/bjoc.13.250

**Published:** 2017-11-29

**Authors:** Sergey L Deev, Alexander S Paramonov, Tatyana S Shestakova, Igor A Khalymbadzha, Oleg N Chupakhin, Julia O Subbotina, Oleg S Eltsov, Pavel A Slepukhin, Vladimir L Rusinov, Alexander S Arseniev, Zakhar O Shenkarev

**Affiliations:** 1 Ural Federal University, 19 Mira Street, 620002 Yekaterinburg, Russia; 2 Shemyakin-Ovchinnikov Institute of Bioorganic Chemistry, Russian Academy of Sciences, 16/10 Miklukho-Maklaya Street, 117997 Moscow, Russia; 3 I. Ya. Postovsky Institute of Organic Synthesis, Ural Branch of the Russian Academy of Sciences, 22 S. Kovalevskoy Street, 620219 Yekaterinburg, Russia

**Keywords:** adamantylation, azolo-1,2,4-triazines, *J*-coupling, ^15^N-labelled, NMR spectra, 1,2,4-triazolo[1,5-*a*]pyrimidines

## Abstract

Determining the accurate chemical structures of synthesized compounds is essential for biomedical studies and computer-assisted drug design. The unequivocal determination of N-adamantylation or N-arylation site(s) in nitrogen-rich heterocycles, characterized by a low density of hydrogen atoms, using NMR methods at natural isotopic abundance is difficult. In these compounds, the heterocyclic moiety is covalently attached to the carbon atom of the substituent group that has no bound hydrogen atoms, and the connection between the two moieties of the compound cannot always be established via conventional ^1^H-^1^H and ^1^H-^13^C NMR correlation experiments (COSY and HMBC, respectively) or nuclear Overhauser effect spectroscopy (NOESY or ROESY). The selective incorporation of ^15^N-labelled atoms in different positions of the heterocyclic core allowed for the use of ^1^H-^15^N (*J*_HN_) and ^13^C-^15^N (*J*_CN_) coupling constants for the structure determinations of N-alkylated nitrogen-containing heterocycles in solution. This method was tested on the N-adamantylated products in a series of azolo-1,2,4-triazines and 1,2,4-triazolo[1,5-*a*]pyrimidine. The syntheses of adamantylated azolo-azines were based on the interactions of azolo-azines and 1-adamatanol in TFA solution. For azolo-1,2,4-triazinones, the formation of mixtures of *N*-adamantyl derivatives was observed. The *J*_HN_ and *J*_CN_ values were measured using amplitude-modulated 1D ^1^H spin-echo experiments with the selective inversion of the ^15^N nuclei and line-shape analysis in the 1D ^13^С spectra acquired with selective ^15^N decoupling, respectively. Additional spin–spin interactions were detected in the ^15^N-HMBC spectra. NMR data and DFT (density functional theory) calculations permitted to suggest a possible mechanism of isomerization for the adamantylated products of the azolo-1,2,4-triazines. The combined analysis of the *J*_HN_ and *J*_CN_ couplings in ^15^N-labelled compounds provides an efficient method for the structure determination of N-alkylated azolo-azines even in the case of isomer formation. The isomerization of adamantylated tetrazolo[1,5-*b*][1,2,4]triazin-7-ones in acidic conditions occurs through the formation of the adamantyl cation.

## Introduction

**Figure 1:**
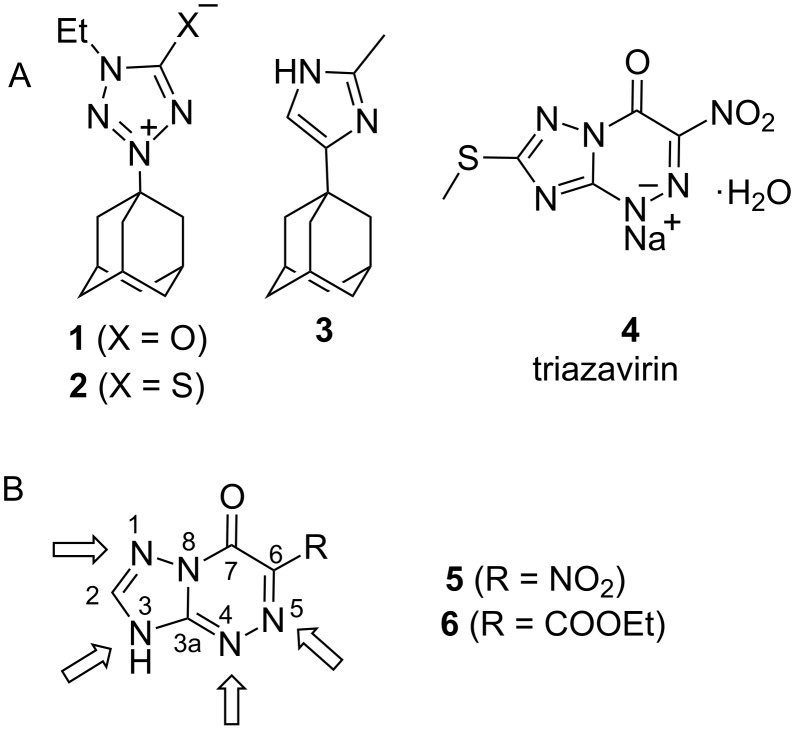
(A) Adamantylated azoles and derivatives of 1,2,4-triazolo[5,1-*c*][1,2,4]triazine with antiviral activities. (B) Four sites sensitive to N-alkylation in 1,2,4-triazolo[5,1-*c*][1,2,4]triazin-7-ones are indicated by arrows.

The incorporation of an adamantyl moiety in bioactive molecules and analogues of natural compounds is a widely used approach in medicinal chemistry [1]. The increased lipophilicity of adamantane-containing compounds compared with non-adamantylated derivatives [2] leads to considerably higher solubility of these compounds in blood plasma and their easier penetration through cell membranes. The conjugation of adamantane with heterocyclic compounds also provides a method to modify the pharmacological profile and frequently leads to a new type of bioactivity. For example, *N*-adamantyl tetrazoles **1** and **2** (Figure 1A) demonstrate lower toxicity and, simultaneously, more potent activity against influenza A virus compared with the currently used antiviral drug rimantadine (1-(1-adamantyl)ethanamine) [3]. More recently, Roberge et al. described new inhibitors of the influenza A virus M2 proton channel. Among the studied compounds, adamantyl imidazole **3** showed good activity [4].

An azolo-azine core with a bridgehead nitrogen atom is found in many natural products [5–6] and biologically active synthetic compounds [7–8]. The purine-like scaffold of these nitrogen-containing heterocycles is frequently used in medicinal chemistry and drug design. For example, 6-nitro-1,2,4-triazolo[5,1-*c*][1,2,4]triazine **4** (Figure 1A, Triazavirin^®^) was approved in Russia for the treatment of influenza [9]. This drug targets the viral protein haemagglutinin. The incorporation of an adamantyl moiety in azolo-azine structures could lead to the development of new multifunctional antiviral drugs.

Previously, we synthesized N-adamatylated derivatives of 1,2,4-triazolo[5,1-*c*][1,2,4]triazines **5** and **6** by reaction with the adamantyl cation generated from 1-adamantanol in acidic medium [10]. The azolo-azine scaffold of these compounds has several nitrogen atoms that can react with alkylation reagents [11–12] (Figure 1B). For this reason, the adamantylation of compounds **5** and **6** led to mixtures of N3- and N4-adamantylated isomers, which reisomerized into each other likely via the formation of an adamantyl cation and starting NH-heterocycle. The unambiguous determination of N-adamantylation site(s) in heterocycles **5** and **6** using well-established ^1^H and ^13^C NMR methods (such as 1D, 2D COSY, HMQC, HMBC, and INADEQUATE spectra) was difficult because the heterocyclic moiety was covalently attached to the adamantane tertiary carbon that had no bound hydrogen atoms. Nuclear Overhauser effect spectroscopy (NOESY or ROESY) also did not provide unequivocal structures of the N-adamantylated derivatives [13–14]. For example, the attachment of an adamantyl group to the N1 or N3 atom in the azole ring of compounds **5** and **6** could not be distinguished by NOE data. Similar problems with the unambiguous determination of the product structure were also found for N-arylation or N-alkylation with *tert*-butyl fragments in the series of 1,2,3-triazole [15–16], tetrazole [17–20], and purine [21] derivatives. Meanwhile, knowledge of the accurate chemical structures of N-substituted heterocycles is essential for biomedical studies and computer-assisted drug design, e.g., molecular docking techniques. Thus, the development of effective methods for the unambiguous determination of N-alkylation site(s) in the azolo-azine series is important.

The data that are required to solve this problem could be provided by ^15^N NMR spectroscopy. For monocyclic derivatives of azoles, the structures of N-alkylated regioisomers can be determined using 2D H-(C)-N multiple bond correlation (HCNMBC) experiments [22–23] using natural isotopic abundance. These experiments rely on the magnetization transfer through ^13^C-^15^N *J*-coupling constants (*J*_CN_). However, the fusion of the azine ring to an azolo fragment increases the number of possible alkylation sites and considerably complicates the analysis of the *J*_CN_ patterns. This issue, together with the inherently low sensitivity of natural abundance ^15^N NMR spectroscopy, does not always permit the unambiguous positioning of alkyl (*N*-adamantyl, *N*-*tert*-butyl or *N*-aryl) fragments in azolo-azines.

The incorporation of ^15^N labels in nitrogen-containing heterocycles greatly facilitates the use of NMR spectroscopy for studies of molecular structures and mechanisms of chemical transformations [10,24–29]. The labelling enhances the sensitivity of detection and permits the quantitative measurements of *J*_CN_ and ^1^H-^15^N *J*-coupling constants (*J*_HN_) even in a mixture of tautomeric forms [24–25]. Additionally, a method based on amplitude-modulated spin-echo experiments was found to be the most efficient way to measure *J*_HN_ couplings [24]. Previously, the incorporation of a single ^15^N label in position 1 of the 1,2,4-triazole fragment of compounds **5** and **6** and analysis of the *J*_CN_ couplings permitted the unambiguous identification of the structures of the N3-adamantylated derivatives (Figure 1B), while the structures of the N4-adamantylated products were determined by ^13^C NMR spectroscopy via comparison with model compounds, N-methylated azolo-azines [10]. However, this preliminarily study did not evaluate the potential of the incorporation of several ^15^N-labels and simultaneous analysis of the *J*_CN_ and *J*_HN_ coupling constants for the determination of the N-adamantylation site(s) in heterocycles.

Herein, we report the selective incorporation of two ^15^N-labelled atoms in tetrazolo[1,5-*b*][1,2,4]triazin-7-one, 1,2,4-triazolo[5,1-*c*][1,2,4]triazin-7-one, and 1,2,4-triazolo[1,5-*a*]pyrimidin-7-one and the N-adamantylation of the obtained compounds. The combined analysis of the *J*_CN_ and *J*_HN_ couplings permitted the straightforward determination of the adamantylation sites in these azolo-azines, even when a mixture of regioisomers is formed.

## Results

**Scheme 1:**
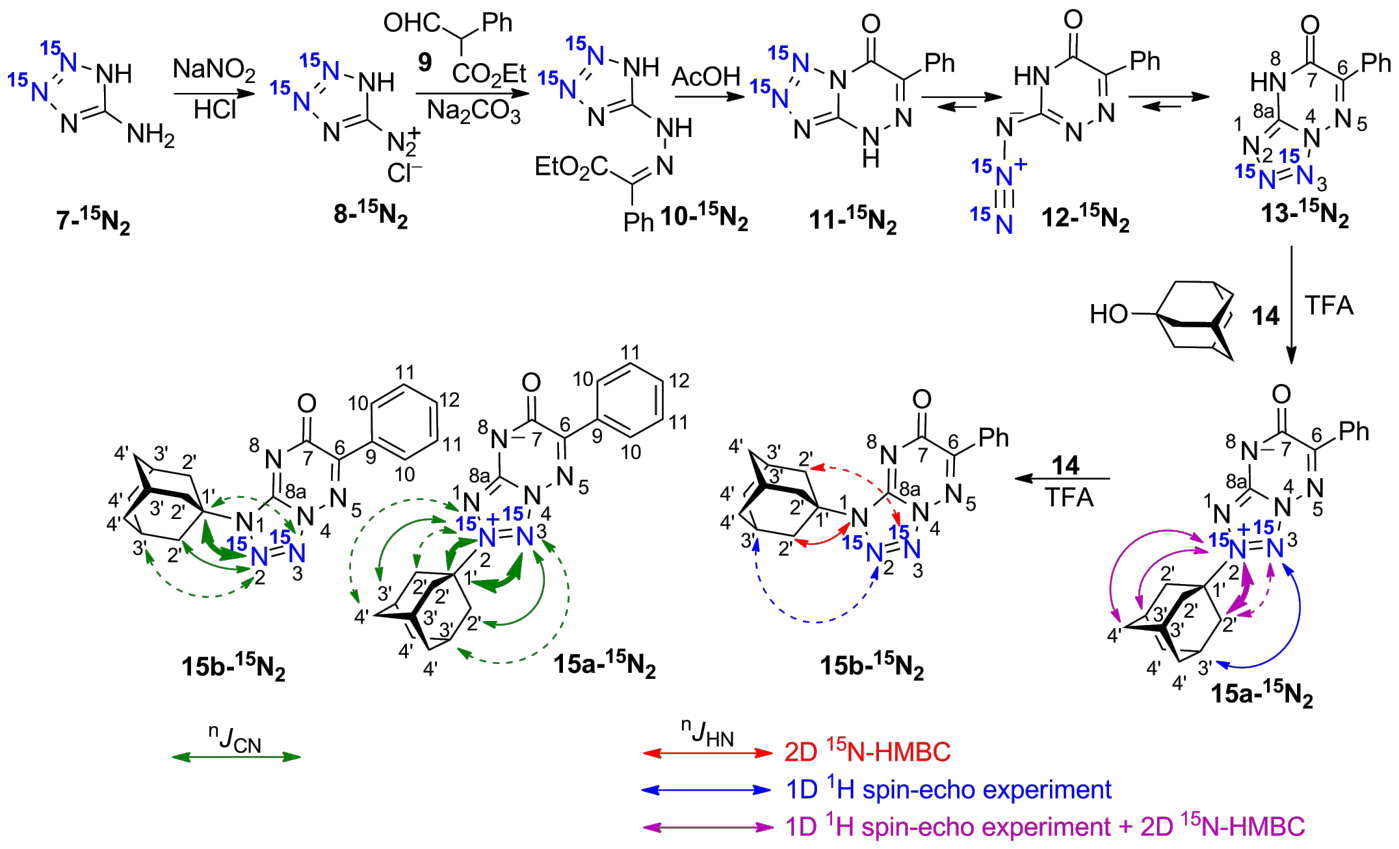
Synthesis and adamantylation of ^15^N-labelled **13-^15^N_2_** and *J*_HN_ and *J*_CN_ data confirming the structures of adamantylated derivatives **15a,b-^15^N_2_**. The *J*_HN_ couplings measured by amplitude-modulated 1D ^1^H spin-echo experiments and detected in the 2D ^15^N-HMBC spectra are shown by blue, magenta, and red arrows (see the legend in the figure). The measured *J*_HN_ values (blue and magenta) are classified into three categories: *J* ≥ 0.8 Hz, 0.8 > *J* ≥ 0.1 Hz, and *J* < 0.1 Hz (bold solid, thin solid, and dashed arrows, respectively). The ^1^H-^15^N cross-peaks observed in the 2D HMBC spectrum for the unlabelled and labelled nitrogen atoms are classified into three categories: strong, medium and weak (bold solid, thin solid, and dashed red arrows, respectively). The *J*_CN_ couplings with adamantane carbons measured in the 1D ^13^С spectra are classified into three categories: *J* ≥ 2 Hz, 2 > *J* ≥ 1 Hz, and *J* < 1 Hz (bold solid, thin solid, and dashed green arrows, respectively). The ^1^*J*_NN_ couplings observed in the 1D ^15^N NMR spectra of **13-^15^N_2_** (16.4 Hz), **15a-^15^N_2_** (14.7 Hz) and **15b-^15^N_2_** (15.3 Hz) are not shown.

**Synthesis.** Derivatives of 1,2,4-triazolo[1,5-*a*]pyrimidine [30], 1,2,4-triazolo[5,1-*c*][1,2,4]triazinone [31] and tetrazolo[1,5-*b*][1,2,4]triazinone [32] can be obtained by the fusion of an azine ring to an azole fragment. This method can be used for the selective incorporation of ^15^N atoms in different azolo-azines. Recently, we tested this approach for the syntheses of ^15^N-labelled tetrazolo[1,5-*b*][1,2,4]triazines and tetrazolo[1,5-*a*]pyrimidines [25] starting from ^15^N-labelled 5-aminotetrazole. However, due to proton tautomerism, the use of single-labelled [2-^15^N]-5-aminotetrazole led to the formation of isotopomer mixtures, which complicated the subsequent NMR analysis. Meanwhile, the application of [2,3-^15^N_2_]-5-aminotetrazole **7-^15^N_2_** provided the single double-labelled products in the tetrazolo[1,5-*a*]pyrimidine series [33]. Thus, in the current work, [2,3-^15^N_2_]-5-aminotetrazole **7-^15^N_2_** (98% enrichment for each of the labelled ^15^N atoms) was used to incorporate isotopic labels in the tetrazolo[1,5-*b*][1,2,4]triazine core (Scheme 1). The interaction of diazonium salt **8-^15^N_2_** derived from [2,3-^15^N_2_]-5-aminotetrazole **7-^15^N_2_** with ethyl α-formylphenylacetate (**9**) yielded compound **10-^15^N_2_**. It was expected that the cyclization of **10-^15^N_2_** would give [1,2-^15^N_2_]-tetrazolo[5,1-*c*][1,2,4]triazine **11-^15^N_2_**. Indeed, [2,3-^15^N_2_]-tetrazolo[1,5-*b*][1,2,4]triazin-7-one **13-^15^N_2_** was obtained (see below). Most likely, tetrazole **11-^15^N_2_** underwent a ring-opening process, yielding azide **12-^15^N_2_**, and this process was followed by an alternative ring closure. This azido-tetrazole equilibrium has been previously studied in detail [25].

The coupling between compound **13-^15^N_2_** and 1-adamantanol (**14**) was conducted in trifluoroacetic acid (TFA) solution under reflux. A general and convenient approach to the N-adamantylation of heterocycles involves a reaction with the adamantyl cation generated from 1-adamantanol in acidic medium [34–37]. The adamantylation of **13-^15^N_2_** led to N2- and N1-regioisomers (**15a-^15^N_2_** and **15b-^15^N_2_**, respectively, Scheme 1). Interestingly, according to the possible resonance structures, compound **15a-^15^N_2_** should represent a mesoionic (betaine-like) structure with positive and negative charges located at the tetrazole and triazine rings, respectively. The relative concentration of regioisomers **15a-^15^N_2_** and **15b-^15^N_2_** was determined from the integral intensity of the corresponding signals in the 1D ^1^H and ^15^N NMR spectra. The regioselectivity of adamantylation depends on the reaction time. Refluxing of the **13-^15^N_2_**/**14** mixture (1:1.5 mol/mol) in TFA over 5 min led to the predominant formation of N2-adamantylated derivative **15a-^15^N_2_**. The 1:2 **15a-^15^N_2_**/**15b-^15^N_2_** mixture was obtained after 2 h of refluxing. This phenomenon could be explained by the reisomerization of the initially formed N2-adamantylated product (**15a-^15^N_2_**). Indeed, 2 h of refluxing of isolated **15a-^15^N_2_** in TFA with 1.5 molar equivalents of **14** yielded a mixture of compounds **15a-^15^N_2_** and **15b-^15^N_2_** in the same (1:2) ratio (Scheme 1).

**Scheme 2:**
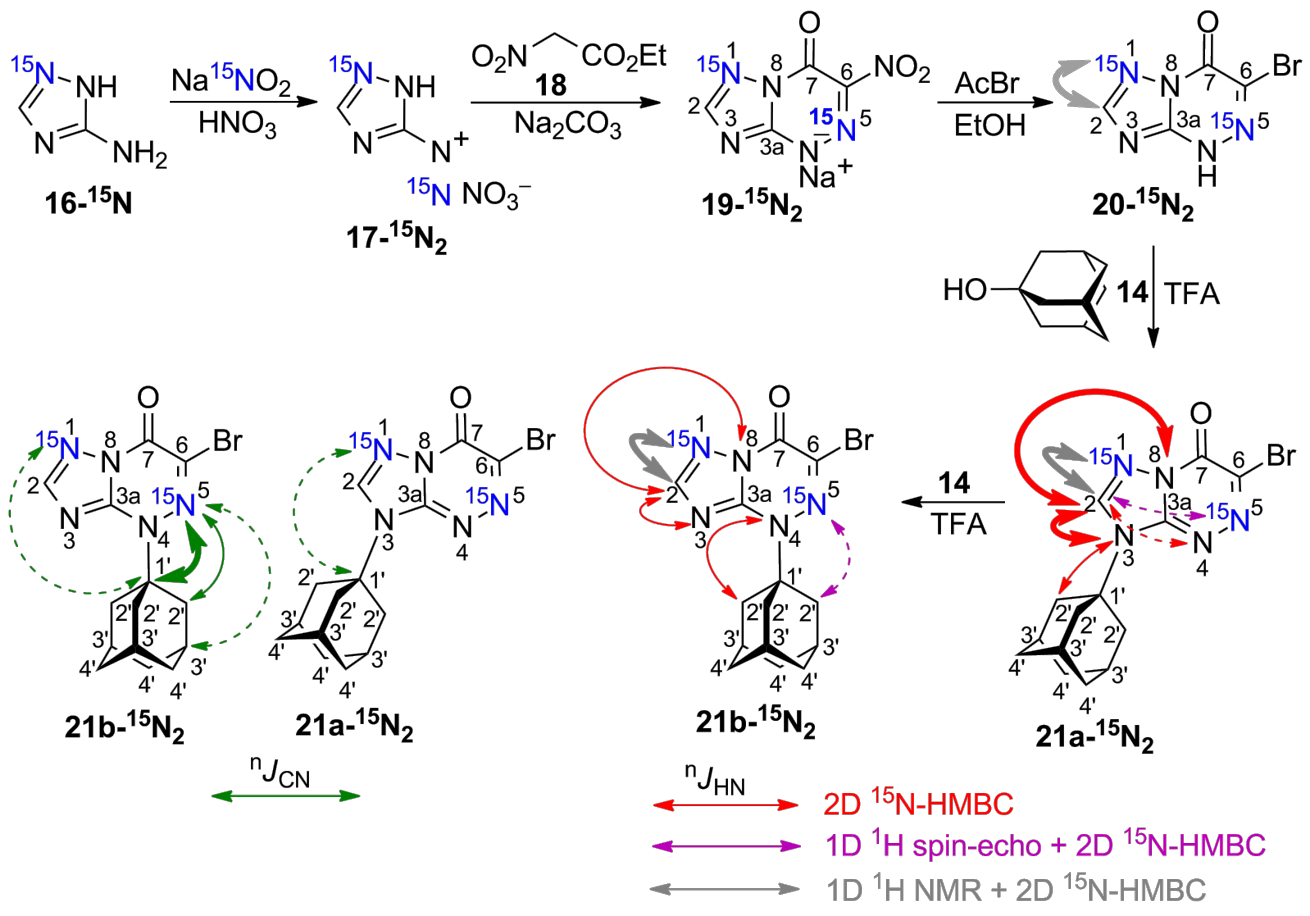
Synthesis and adamantylation of ^15^N-labelled **20-^15^N_2_** and *J*_HN_ and *J*_CN_ data confirming the structures of adamantylated derivatives **21a,b-^15^N_2_**.The *J*_HN_ couplings measured either in the 1D ^1^H spectra or by amplitude-modulated 1D ^1^H spin-echo experiments and detected in the 2D ^15^N-HMBC spectra are shown by grey, magenta, and red arrows (see the legend in the figure). The measured *J*_HN_ values (gray and magenta) with magnitudes *J* ≥ 14 Hz and *J* < 0.1 Hz are indicated by the bold solid and dashed arrows, respectively. The ^1^H-^15^N cross-peaks observed in the 2D HMBC spectrum for the unlabelled nitrogen atoms are classified into three categories: strong, medium and weak (bold solid, thin solid, and dashed red arrows, respectively). The *J*_CN_ couplings with adamantane carbons measured in the 1D ^13^С spectra are classified into three categories: *J* ≥ 2 Hz, 2 > *J* ≥ 1 Hz, and *J* < 1 Hz (bold solid, thin solid, and dashed green arrows, respectively).

The use of [1-^15^N]-3-amino-1,2,4-triazole **16-^15^N** (98%, ^15^N) and labelled sodium nitrite (98%, ^15^N) in acidic medium allowed for the in situ production of diazonium salt **17-^15^N_2_**, which reacted with ethyl nitroacetate (**18**) in a sodium carbonate solution (Scheme 2). This reaction led to the formation of [1,5-^15^N_2_]-1,2,4-triazolo[5,1-*c*][1,2,4]triazinone **19-^15^N_2_**. Previously, the same approach was described for the incorporation of ^15^N atoms in azole and azine rings of compound **4** [38]. Heterocycle **20-^15^N_2_** was obtained by the treatment of **19-^15^N_2_** with hydrobromic acid according to a procedure described for 6-nitro-1,2,4-triazolo[5,1-*c*][1,2,4]triazin-7-ones [39]. In this case, hydrogen bromide was obtained in situ by the reaction between acetyl bromide and ethanol. The adamantylation of **20-^15^N_2_** first occurred on the N3 atom of the azole ring. It was found that 5 min reflux of **20-^15^N_2_** in TFA with a 1.5 molar excess of **14** led to the structure **21a-^15^N_2_** (Scheme 2). However, prolonged (6 h) refluxing of the N3-regioisomer with 1.5 molar equivalents of 1-adamantanol (**14**) in a TFA solution led to complete isomerization of the compound and re-attachment of adamantane to the N4-atom of the azine ring (compound **21b-^15^N_2_**).

**Scheme 3:**
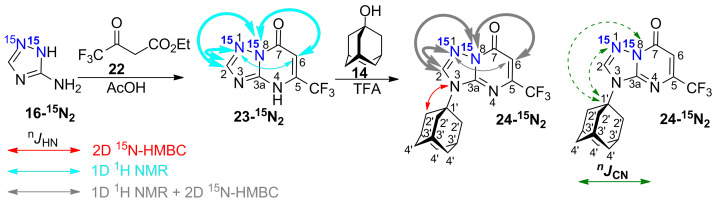
Synthesis and adamantylation of ^15^N-labelled **23-^15^N_2_** and *J*_HN_ and *J*_CN_ data confirming the structure of adamantylated derivative **24-^15^N_2_**. The *J*_HN_ couplings measured in the 1D ^1^H spectra and detected in the 2D ^15^N-HMBC spectra are shown by grey, cyan, and red arrows (see the legend in the figure). The measured *J*_HN_ values (gray and cyan) with magnitudes *J* ≥ 3 Hz and *J* < 1 Hz are indicated by the bold and thin solid arrows, respectively. The correlation between H2' and the unlabelled N3 atom observed in the HMBC spectrum of **24-^15^N_2_** is shown by a red arrow. The *J*_CN_ couplings with the H1' adamantane carbon measured in the 1D ^13^С spectrum of **24-^15^N_2_** have magnitudes < 1 Hz and are shown by dashed green arrows. The ^1^*J*_NN_ couplings observed in the 1D ^15^N NMR spectra of **23-^15^N_2_** (13.6 Hz) and **24-^15^N_2_** (13.4 Hz) are not shown.

Double-labelled [1,2-^15^N_2_]-3-amino-1,2,4-triazole **16-^15^N_2_** was synthesized by the interaction of ^15^N_2_-hydrazine sulphate (98%, ^15^N) with *S*-methyl isothiourea sulphate and consecutive cyclization with formic acid (see the Supporting Information File 1). The use of **16-^15^N_2_** in a reaction with ethyl 4,4,4-trifluoroacetoacetate (**22**) yielded azolo-azine **23-^15^N_2_** containing two isotopic labels in the 1,2,4-triazole fragment (Scheme 3). The adamantylation of [1,8-^15^N_2_]-1,2,4-triazolo[1,5-*a*]pyrimidine **23-^15^N_2_** was regioselective and led to the formation of the N3-isomer **24-^15^N_2_** only. This compound did not undergo further isomerization.

**Isomerization of adamantylated derivatives.** The adamantylation of 1,2,4-triazolo[5,1-*c*][1,2,4]triazin-7-one derivatives in acidic medium is a thermodynamically controlled reaction [10], which could explain the rearrangement of N3-isomer **21a** into N4-isomer **21b** and the formation of the **15a**/**15b** mixture from compound **15a**. To evaluate the relative thermodynamic stabilities of isomers **15a,b** and **21a,b**, we performed DFT calculations with the RB3LYP/6-31-G(d,p) approximation in the gas phase using the Gaussian 09 package [40]. Isomers **15b** and **21b** are thermodynamically more stable than counterparts **15a** and **21a**. The calculated relative energy differences were 8.3 kcal/mol and 6.4 kcal/mol for the **15a–15b** and **21a–21b** pairs, respectively (see the Supporting Information File 1).

**Scheme 4:**
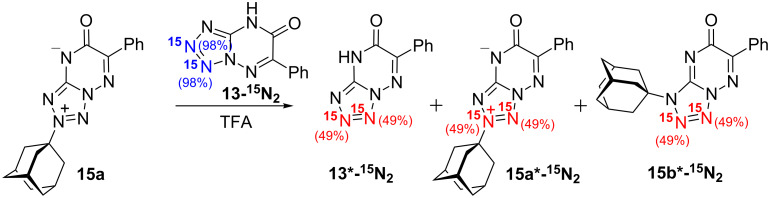
Isomerization of **15a** in the presence of tetrazolo[1,5-*b*][1,2,4]triazin-7-one **13-^15^N_2_** and isotopic enrichment of the reactants and products. The starting level of ^15^N-isotopic enrichment (98%) of compound **13-^15^N_2_** is shown in blue. The levels of ^15^N enrichment (≈49%) of the obtained compounds 13*-^15^N_2_, 15a*-^15^N_2_ and 15b*-^15^N_2_ are shown in red. The levels of isotopic enrichment were determined by mass spectrometry. In addition, ≈50% excess of the ^15^N isotopes in compounds 15a*-^15^N_2_ and 15b*-^15^N_2_ after reaction was confirmed by ^13^C NMR spectroscopy. In this case, the C1' signals of the labelled and unlabelled components demonstrated approximately equal integral intensities (Figure S24 in Supporting Information File 1).

To further study the mechanism of isomerization between compounds **15a** and **15b**, equimolar quantities of unlabelled N2-isomer **15a** and its double-labelled non-adamantylated precursor **13-^15^N_2_** (isotopic enrichment 98%) were refluxed for 2 h in TFA without the addition of 1-adamantanol (**14**, Scheme 4). NMR analysis of the resulting mixture revealed the compounds 13*-^15^N_2_, 15a*-^15^N_2_, and 15b*-^15^N_2_ in a 5:2:3 ratio. The observed equal ^15^N-isotopic enrichment (≈49%) in compounds 13*-^15^N_2_, 15a*-^15^N_2_, and 15b*-^15^N_2_ indicated that the equilibrium **15a**
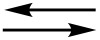
**15b** was reached in the isomerization process. The obtained ratio between the adamantylated products confirmed the higher thermodynamic stability of compound **15b** relative to isomer **15a**.

**Figure 2:**
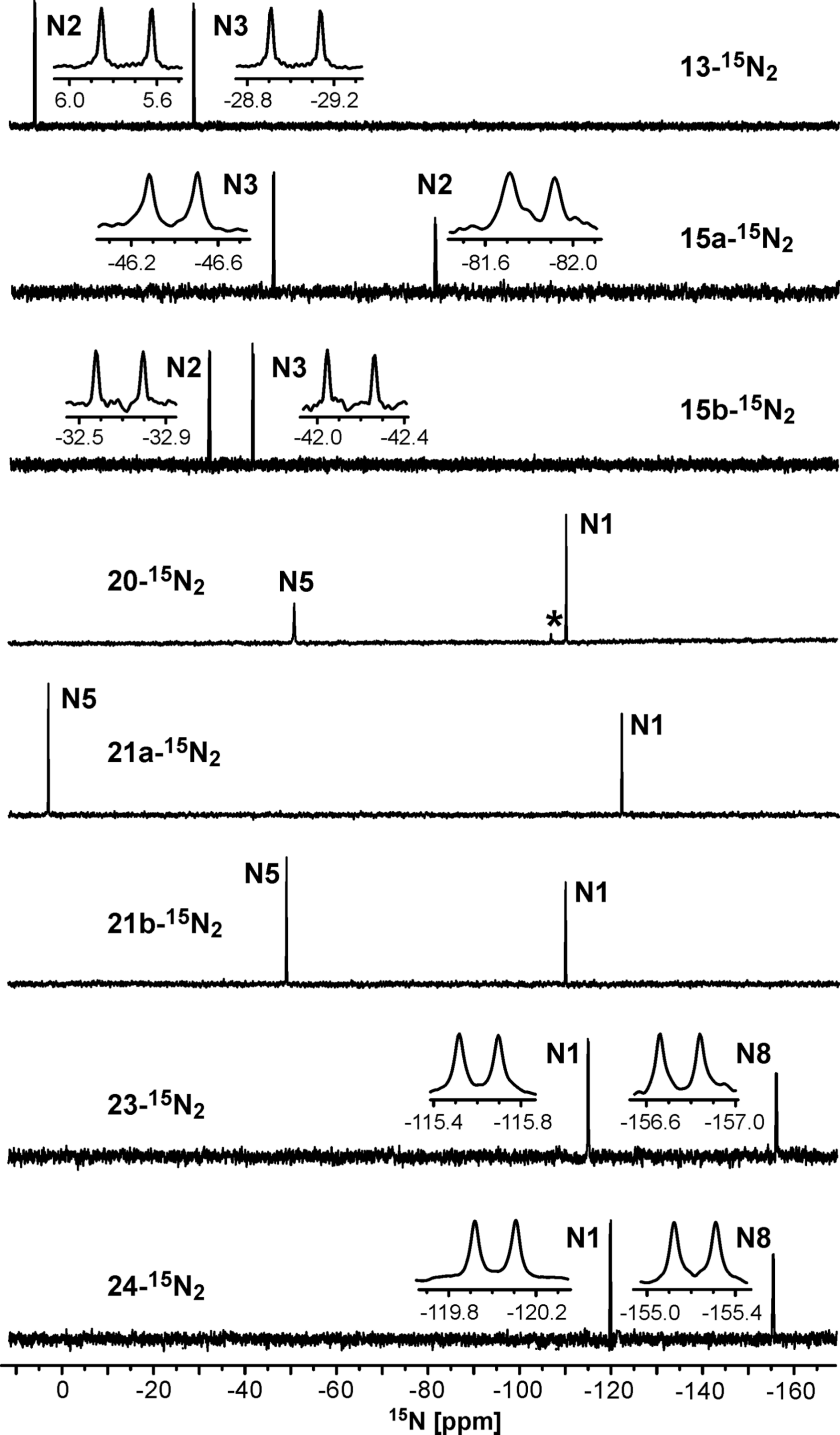
1D ^15^N NMR spectra of 30–70 mM **13-^15^N_2_**, **15a,b-^15^N_2_**, **20-^15^N_2_**, **21a,b-^15^N_2_**, **23-^15^N_2_** and **24-^15^N_2_** in DMSO-*d*_6_ (45 °C). The signal of the impurity (a base formed from salt **19-^15^N_2_** in acidic medium) is marked by an asterisk.

**NMR spectroscopy and resonance assignment.** The synthesized compounds were studied by NMR spectroscopy in a dimethyl sulfoxide (DMSO-*d*_6_) solution using samples with concentrations with range of 30–70 mM. The obtained 1D ^15^N NMR spectra are shown in Figure 2, and the 1D ^1^H and ^13^C spectra are presented as Figures S1–S18 in Supporting Information File 1. Two signals corresponding to the ^15^N-labelled atoms were observed in the 1D ^15^N NMR spectra of all the starting azolo-azines and adamantylated products (Figure 2). The ^15^N spectra of compounds **13-^15^N_2_**, **15a,b-^15^N_2_**, **23-^15^N_2_** and **24-^15^N_2_** containing labelled nitrogens in the neighbouring positions also demonstrated 13.4–16.4 Hz splittings due to the direct ^1^*J*_NN_ coupling constants (Figure 2, Table 1).

**Table 1:** ^1^H and ^15^N chemical shifts (ppm), ^1^H-^15^N and ^15^N-^15^N *J*-coupling constants (Hz), and ^1^H-^15^N spin–spin interactions observed in the 2D ^15^N-HMBC spectra of the synthesized compounds.

compound	δ(^15^N)^a^, *J*_NN_^b^, *J*_HN_^c^ and ^15^N-HMBC peaks^d^			δ(^1^H)^e^	

	^15^N-labelled N2/N1	^15^N-labelled N3/N5/N8	^15^N at natural abundance	Ad	H2,H6/Ph
	
**13-^15^N_2_**	5.74 (N2) ^1^*J*_N2-N3_ 16.4	−29.02 (N3) ^1^*J*_N3-N2_ 16.4			8.036 (H10) 7.563 (H11) 7.622 (H12)
**15a-^15^N_2_**	−81.81 (N2) ^1^*J*_N2-N3_ 14.7 ^3^*J*_H2'-N2_ 0.83 (s) ^4^*J*_H3'-N2_ 0.60 (m) ^5^*J*_H4'-N2_ 0.23 (m)	−46.42 (N3) ^1^*J*_N3-N2_ 14.7 ^4^*J*_H2'-N3_ 0.06 (w) ^5^*J*_H3'-N3_ 0.11 (–)		2.361 (H2')^f^ 2.306 (H3') 1.792 (H4')	8.141 (H10) 7.535 (H11) 7.597 (H12)
**15b-^15^N_2_**	−32.69 (N2) ^1^*J*_N2-N3_ 15.3 ^4^*J*_H2'-N2_ < 0.04^g^ (–) ^5^*J*_H3'-N2_ 0.04 (–)	−42.14 (N3) ^1^*J*_N3-N2_ 15.3 ^5^*J*_H2'-N3_ < 0.04^g^ (w)	−159.34 (N1) ^3^*J*_H2'-N1_ (m)	2.412 (H2')^f^ 2.258 (H3') 1.776 (H4')	8.088 (H10) 7.538 (H11) 7.589 (H12)
**19-^15^N_2_**	−108.06 (N1) ^2^*J*_H2-N1_ 15.9^h^	19.27 (N5)			8.309 (H2)
**20-^15^N_2_**	−110.79 (N1) ^2^*J*_H2-N1_ 16.1^h^	−51.36 (N5)			8.359 (H2)
**21a-^15^N_2_**	−122.55 (N1) ^2^*J*_H2-N1_ 14.0^h^ (s)	2.79 (N5) ^5^*J*_H2-N5_ 0.07 (w)	−212.12 (N3) ^3^*J*_H2'-N3_ (m) ^3^*J*_H2-N3_ (s) −249.60 (N4) ^4^*J*_H2-N4_ (w) −163.08 (N8) ^3^*J*_H2-N8_ (s)	2.390 (H2')^f^ 2.231 (H3') 1.752 (H4')	9.039 (H2)
**21b-^15^N_2_**	−110.24 (N1) ^2^*J*_H2-N1_ 16.0^h^ (s)	−49.26 (N5) ^4^*J*_H2'-N5_ 0.06 (w)	159.08 (N3) ^2^*J*_H2-N3_ (m) −192.59 (N4) ^3^*J*_H2'-N4_ (m) −155.08 (N8) ^3^*J*_H2-N8_ (m)	2.392 (H2')^f^ 2.241 (H3') 1.736 (H4')	8.409 (H2)
**23-^15^N_2_**	−115.62 (N1) ^1^*J*_N1-N8_ 13.6 ^2^*J*_H2-N1_ 14.5^h^ ^4^*J*_H6-N1_ 0.8^h^	−156.75 (N8) ^1^*J*_N8-N1_ 13.6 ^3^*J*_H2-N8_ 6.4^h^ ^3^*J*_H6-N8_ 3.5^h^			8.945 (H2) 6.482 (H6)
**24-^15^N_2_**	−120.03 (N1) ^1^*J*_N1-N8_ 13.4 ^2^*J*_H2-N1_ 13.8^h^ (s) ^4^*J*_H6-N1_ 0.9^h^ (m)	−155.21 (N8) ^1^*J*_N8-N1_ 13.4 ^3^*J*_H2-N8_ 6.6^h^ (s) ^3^*J*_H6-N8_ 3.4^h^ (s)	−206.85 (N3) ^3^*J*_H2'-N3_ (m)	2.373 (H2')^f^ 2.210 (H3') 1.729 (H4')	8.996 (H2) 6.521 (H6)

^a^The ^15^N chemical shifts were referenced indirectly relative to MeNO_3_. The ^15^N-signals of the labelled atoms were observed in the 1D ^15^N NMR spectra, and the ^15^N-signals at natural isotopic abundance were observed in the 2D ^15^N-HMBC spectra. ^b^The *J*_NN_ coupling constants were measured in the 1D ^15^N NMR spectra. The estimated error in the *J*_NN_ values is ≈0.1 Hz. ^c^Unless otherwise stated, the *J*_HN_ values were measured using amplitude-modulated 1D ^1^H spin-echo experiments with delays for the evolution of *J*_HN_ up to 1 s. The estimated error in the *J*_HN_ values is 0.02 Hz, and the lower limit of reliable *J*_HN_ measurements is 0.04 Hz. ^d^The cross-peaks in the 2D ^15^N-HMBC spectra were classified into three categories (weak – w; medium – m; strong – s). Weak peaks approximately correspond to *J*_HN_ < 0.5 Hz, strong peaks approximately correspond to *J*_HN_ > 2 Hz and medium peaks correspond to the other values. The degree of isotopic enrichment was accounted for. It was assumed that the intensity of the HMBC cross-peak is proportional to the sin^2^(π·*J*_HN_·Δ), where Δ is the delay used for the magnetization transfer (62–125 ms). (–) Indicates unobserved HMBC cross-peaks. ^e^The ^1^H chemical shifts were referenced relative to the residual signal of DMSO-*d*_6_ at 2.50 ppm. ^f^The signal demonstrated additional splitting, which is likely related to the slow exchange between the rotamers of adamantane substituents (see text for details). ^g^The measurement of the *J*_HN_ values was impossible due to the fast transverse relaxation of the corresponding ^1^H nuclei. ^h^The *J*_HN_ coupling constants were measured in the 1D ^1^H NMR spectra. The estimated error is 0.1 Hz.

The assignments of the ^13^C and ^15^N signals in the synthesized compounds were obtained by analysing the 2D ^13^C-HMQC, ^13^C-HMBC and ^15^N-HMBC spectra and observing the ^13^C-^15^N and ^1^H-^15^N spin–spin interactions (see below). The ^13^C assignment procedure for **19-^15^N_2_**, **20-^15^N_2_**, and **21a,b-^15^N_2_** was aided by the data from a previous study of unlabelled derivatives of compound **19** [12]. The ^13^C-^19^F *J*-coupling constants (*^n^J*_CF_, Table 2) observed in the 1D ^13^C spectra facilitated the assignment of the ^13^C nuclei for the heterocyclic moieties of compounds **23-^15^N_2_** and **24-^15^N_2_**. The observations of the ^3^*J*_H2-C3a_ coupling constants (9.2 Hz) in the 1D ^13^C spectra of **19-^15^N_2_** and **20-^15^N_2_** measured without proton decoupling confirmed the assignment of C3a to the signals at 160.23 ppm and 152.32 ppm, respectively. The obtained NMR assignments are collected in Table 1 (^1^H, ^15^N) and Table 2 (^13^C).

**Table 2:** ^13^С Chemical shifts (ppm) and ^1^H-^13^C, ^13^C-^15^N and ^13^C-^19^F *J*-coupling constants (Hz) of the studied compounds^a^.

compound	C2/Ph	C3a/C8a	C6	C7	Ad, C5, CF_3_	

**13-^15^N_2_**	131.35 (C9) 129.99 (C10) 128.76(C11) 132.01 (C12)	145.99 (C8a) ^2^*J*_C-N2_ 2.0 ^2^*J*_C-N3_ 3.3	152.15 ^4^*J*_C-N2_ 0.8 ^3^*J*_C-N3_ 1.5	154.31 ^4^*J*_C-N2_ 0.2		
**15a-^15^N_2_**	132.70 (C9) 130.12 (C10) 128.58 (C11) 131.85 (C12)	154.61 (C8a) ^2^*J*_C-N2_ 0.9 ^2^*J*_C-N3_ 2.4	154.47 ^4^*J*_C-N2_ 0.6 ^3^*J*_C-N3_ 1.1	161.03 ^4^*J*_C-N3_ 0.3	69.26 (C1')^b^ ^1^*J*_C-N2_ 6.5 ^2^*J*_C-N3_ 3.8 29.44 (C3') ^3^*J*_C-N2_ 1.6 ^4^*J*_C-N3_ 0.2	41.30 (C2')^b^ ^2^*J*_C-N2_ 0.4 ^3^*J*_C-N3_ 1.2 35.39 (C4') ^4^*J*_C-N2_ 0.3
**15b-^15^N_2_**	132.47 (C9) 129.86 (C10) 128.65 (C11) 131.62 (C12)	144.56 (C8a) ^2^*J*_C-N2_ 0.7	151.04 ^4^*J*_C-N2_ 0.8 ^3^*J*_C-N3_ 1.8	160.72 ^4^*J*_C-N2_ 0.2	63.52 (C1')^b^ ^2^*J*_C-N2_ 2.7 ^3^*J*_C-N3_ 0.3 29.30 (C3') ^4^*J*_C-N2_ 0.3	39.93 (C2')^b,c^ ^3^*J*_C-N2_ 1.1 35.67 (C4')
**19-^15^N_2_**	154.98 (C2) ^1^*J*_C-N1_ 3.7 ^4^*J*_C-N5_ 0.2 ^1^*J*_H2-C_ 206.9^d^	160.23 (C3a) ^2^*J*_C-N1_ 0.3 ^2^*J*_C-N5_ 2.0 ^3^*J*_H2-C_ 9.2^d^	144.84^e,f^	144.41 ^2^*J*_C-N1_ 3.6 ^2^*J*_C-N5_ 1.3		
**20-^15^N_2_**	154.23 (C2) ^1^*J*_C-N1_ 3.3 ^1^*J*_H2-C_ 211.0^d^	152.32 (C3a) ^2^*J*_C-N5_ 2.3 ^3^*J*_H2-C_ 9.2^d^	126.82 ^3^*J*_C-N1_ 1.3 ^1^*J*_C-N5_ 1.9	147.62 ^2^*J*_C-N1_ 3.4 ^2^*J*_C-N5_ 1.3		
**21a-^15^N_2_**	142.93 (C2) ^1^*J*_C-N1_ 1.4	149.56 (C3a) ^2^*J*_C-N1_ 1.8 ^2^*J*_C-N5_ 2.8	133.40 ^3^*J*_C-N1_ 1.3 ^1^*J*_C-N5_ 7.3	145.65 ^2^*J*_C-N1_ 3.1 ^2^*J*_C-N5_ 1.4	60.58 (C1')^b^ ^3^*J*_C-N1_ 0.4 29.40 (C3')^b^	40.11 (C2')^b,c^ 35.70 (C4')
**21b-^15^N_2_**	153.34 (C2) ^1^*J*_C-N1_ 3.2	151.10 (C3a) ^2^*J*_C-N1_ ≤ 0.2^f^ ^2^*J*_C-N5_ 2.3	123.80 ^3^*J*_C-N1_ 1.3 ^1^*J*_C-N5_ 2.7	147.04 ^2^*J*_C-N1_ 3.4 ^2^*J*_C-N5_ 1.1	68.15 (C1')^b^ ^4^*J*_C-N1_ ≤ 0.2^f^ ^2^*J*_C-N5_ 5.0 29.89 (C3')^b^ ^4^*J*_C-N5_ 0.4	39.82 (C2')^b,c^ ^3^*J*_C-N5_ 1.7 35.92 (C4')^b^
**23-^15^N_2_**	143.27 (C2) ^1^*J*_C-N1_ 1.4 ^2^*J*_C-N8_ 1.0	151.09 (C3a)^g^ ^2^*J*_C-N1_ 1.8 ^1^*J*_C-N8_ 11.4 ^4^*J*_C-F_ 0.5	100.46 ^3^*J*_C-N1_ 1.1 ^2^*J*_C-N8_ 8.3 ^3^*J*_C-F_ 3.0	155.84 ^2^*J*_C-N1_ 3.0 ^1^*J*_C-N8_ 10.7 ^4^*J*_C-F_ 0.6	151.08 (C5)^g^ ^3^*J*_C-N8_ 1.1 ^2^*J*_C-F_ 34.0	121.63 (CF_3_) ^4^*J*_C-N8_ 0.3 ^1^*J*_C-F_ 275.0
**24-^15^N_2_**	142.18 (C2) ^1^*J*_C-N1_ 1.2 ^2^*J*_C-N8_ 1.2	149.36 (C3a) ^2^*J*_C-N1_ 1.9 ^1^*J*_C-N8_ 12.0 ^4^*J*_C-F_ 0.7	100.88 ^3^*J*_C-N1_ 1.1 ^2^*J*_C-N8_ 8.1 ^3^*J*_C-F_ 2.8	156.04 ^2^*J*_C-N1_ 3.2 ^1^*J*_C-N8_ 10.4 ^4^*J*_C-F_ 0.4	60.58 (C1') ^3^*J*_C-N1_ 0.4 ^3^*J*_C-N8_ 0.6 29.51 (C3') 150.48 (C5) ^3^*J*_C-N8_ 1.2 ^2^*J*_C-F_ 34.1	40.14 (C2')^c^ 35.86 (C4') 121.64 (CF_3_) ^4^*J*_C-N8_ ≤ 0.3^f^ ^1^*J*_C-F_ 275.0

^a^The ^13^C chemical shifts were referenced indirectly relative to tetramethylsilane (TMS). Using this indirect scale, the ^13^C signal of DMSO-*d*_6_ was observed at 40.155 ppm. The ^13^C-^15^N and ^13^C-^19^F *J*-coupling constants (*J*_CN_ and *J*_CF_, respectively) were measured by line-shape analysis in the 1D ^13^С spectra acquired with selective ^15^N decoupling and broadband ^1^H decoupling. The estimated error in the *J*_CN_ values is 0.1 Hz, and the lower limit of reliable *J*_CN_ measurements is 0.2 Hz. ^b^The signal demonstrated additional splitting, which is likely related to slow exchange between the rotamers of the adamantane substituents (see text for details). The fitted intensity ratio for the two components was 10:7. ^с^The signal is overlapped with the ^13^C signal of DMSO-*d*_6_. ^d^The ^1^H-^13^C *J*-coupling constants (*J*_HC_) were measured by line-shape analysis in the 1D ^13^С spectra acquired without ^1^H decoupling. ^e^The signal demonstrated additional broadening, which was not related to the *J*_CN_ couplings. ^f^Precise measurements of *J*_CN_ couplings were impossible due to low intensity of the corresponding ^13^С resonance. ^g^The C5 and C3a signals overlap.

**Figure 3:**
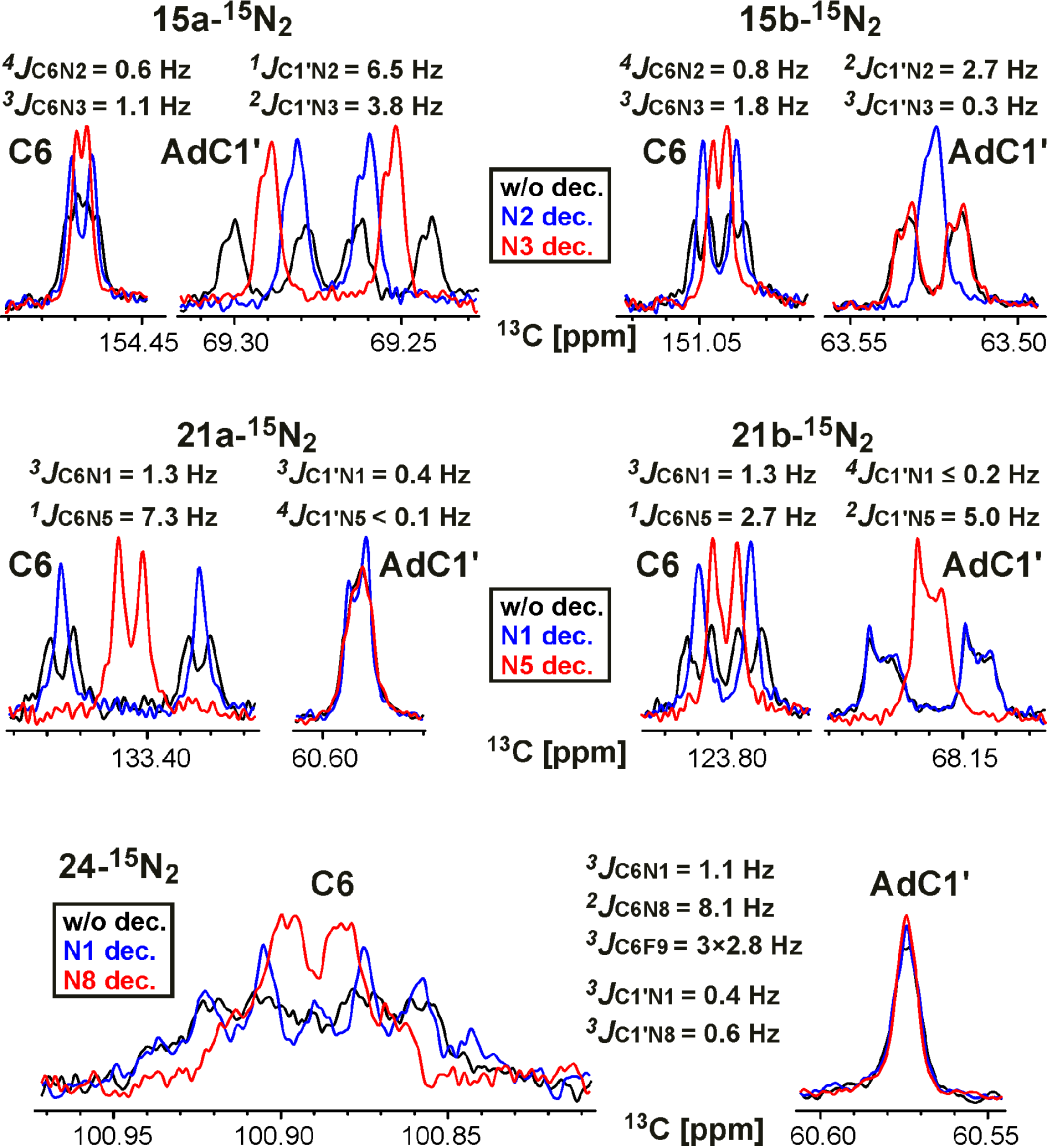
Signals of the C1' and C6 atoms in the proton-decoupled 1D ^13^C NMR spectra of 30–42 mM **15a,b-^15^N_2_, 21a,b-^15^N_2_** and **24-^15^N_2_** in DMSO-*d*_6_ (45 ºC). The spectra were measured without (black traces) and with band-selective decoupling from the ^15^N1/^15^N2 or ^15^N3/^15^N5/^15^N8 nuclei (blue or red traces, respectively). The values of *J*_CN_ obtained by line-shape analysis are listed. The additional splittings of the C1' signals are due to the presence of two structural forms of adamantane substituents (see text for details).

**^13^C-^15^N couplings for the structure determination of N-adamantylated azoloazines.** The incorporation of ^15^N labels into the synthesized compounds led to the appearance of ^1^H-^15^N and ^13^C-^15^N *J*-coupling constants (*J*_CN_ and *J*_HN_ couplings, respectively). The *J*_CN_ couplings became evident from the additional splitting of the corresponding signals in the 1D ^13^C NMR spectra and were measured by nonlinear fits of the ^13^С line shapes in the 1D spectra acquired with band-selective decoupling from ^15^N nuclei [25] (Figure 3). This method allowed for the measurement of the ^13^C-^15^N spin-spin interactions of different magnitudes and ranges starting from the direct ^1^*J*_CN_ couplings (magnitudes of 1.2–12.0 Hz) to long-range ^4^*J*_CN_ couplings (magnitudes of 0.2–0.8 Hz). The full list of measured *J*_CN_ couplings is collected in Table 2. The couplings between adamantane carbons and the nitrogens of the heterocycles are shown in Schemes 1–3.

The *J*_CN_ couplings observed for the C6, C7 and C8a atoms in the heterocyclic moieties of compounds **13-^15^N_2_** and **15a,b-^15^N_2_** confirmed the [1,5-*b*]-type fusion between the azole and azine rings in these structures (Table 2, Figure 3). The observation of the direct ^1^*J*_C1'-N2_ (6.5 Hz) and other ^13^C-^15^N interactions for the C1' (^2^*J*_C-N3_ 3.8 Hz), C2' (^2^*J*_C-N2_ 0.4 Hz and ^3^*J*_C-N3_ 1.2 Hz), C3' (^3^*J*_C-N2_ 1.6 Hz and ^4^*J*_C-N3_ 0.2 Hz) and C4' (^4^*J*_C-N2_ 0.3 Hz) atoms of the adamantane group in **15a-^15^N_2_** indicated that the initial adamantylation of **13-^15^N_2_** underwent a reaction with the N2 atom of the tetrazole ring (Scheme 1). However, the detection of geminal (^2^*J*_C1'-N2_ 2.7 Hz), vicinal (^3^*J*_C2'-N2_ 1.1 Hz and ^3^*J*_C1'-N3_ 0.3 Hz) and long-range (^4^*J*_C3'-N2_ 0.3 Hz) couplings in the 1D ^13^C NMR spectra of **15b-^15^N_2_** revealed the attachment of the adamantane fragment to the N1 atom of the tetrazole ring.

The structures of compounds **19-^15^N_2_**, **20-^15^N_2_**, **21a,b-^15^N_2_**, **23-^15^N_2_** and **24-^15^N_2_** were also confirmed by the measured ^n^*J*_CN_ patterns (Table 2). The presence of characteristic vicinal ^3^*J*_C6-N1_ coupling (magnitudes of 1.1-1.3 Hz) and other coupling constants revealed that the fusions of the triazole rings with the triazine (compounds **19**, **20** and **21**) or pyrimidine rings (compounds **23** and **24**) have [5,1-*c*] or [5,1-*a*] configurations, respectively.

The detection of a single ^3^*J*_C1'-N1_ coupling (0.4 Hz) with the adamantane carbons in compound **21a-^15^N_2_** indicated that the substituent group is attached to the N3 atom of the 1,2,4-triazole ring (Figure 2, Scheme 2). Similarly, the N3-adamantylation in compound **24-^15^N_2_** was characterized by two weak ^3^*J*_C1'-N1/N8_ couplings (0.4/0.6 Hz) detected for the C1' atom (Figure 2, Scheme 3). In contrast, the attachment of the adamantane fragment to the N4 atom of the triazine ring in compound **21b-^15^N_2_** led to a large set of observable *J*_CN_ couplings, including geminal (^2^*J*_C1'-N5_ 5.0 Hz), vicinal (^3^*J*_C2'-N5_ 1.7 Hz) and long-range (^4^*J*_C1'-N1_ ≤ 0.2 Hz and ^4^*J*_C3'-N5_ 0.4 Hz) couplings (Figure 2, Scheme 2).

**Figure 4:**
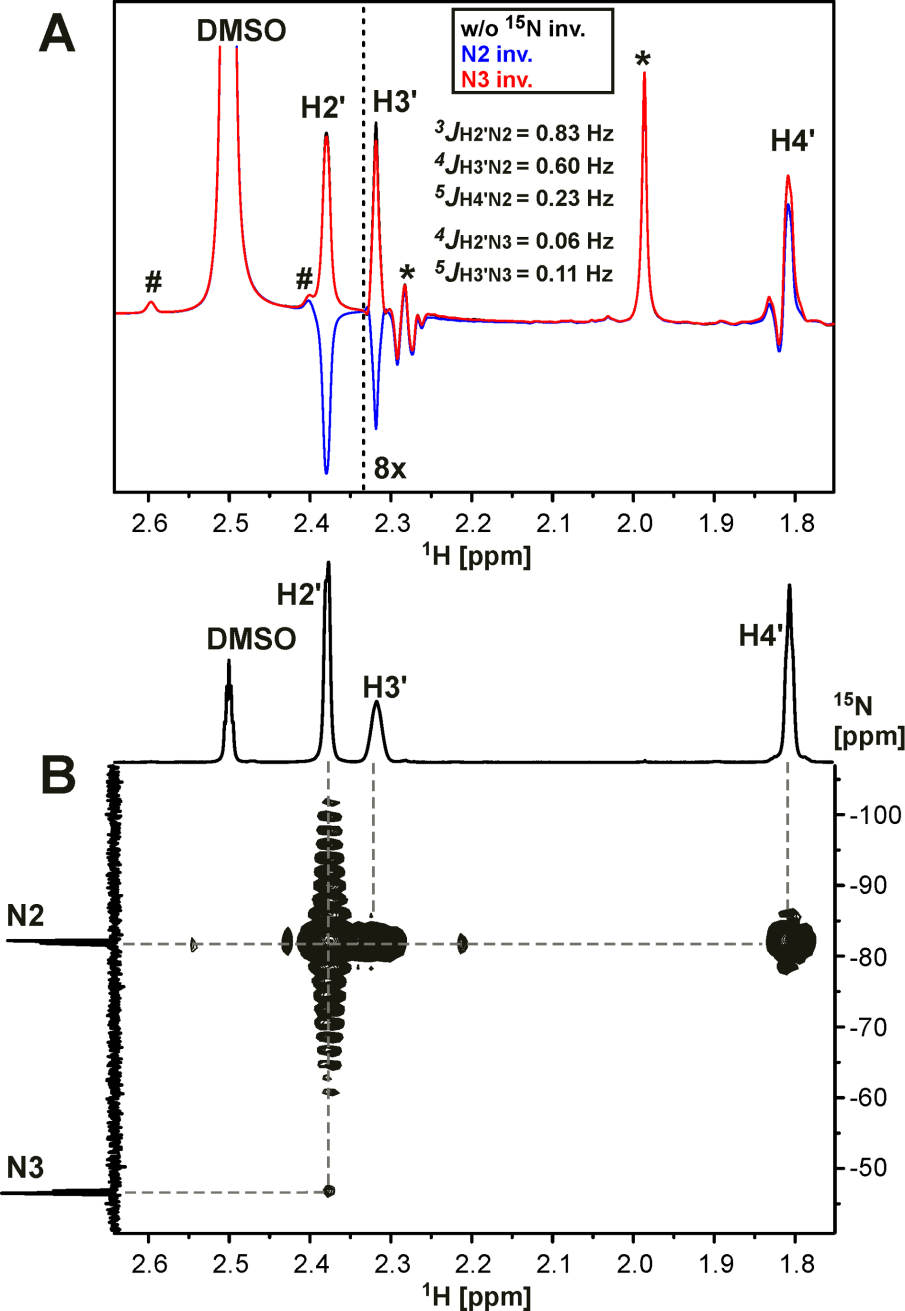
Detection and quantification of the ^1^H-^15^N spin–spin interactions in compound **15a-^15^N_2_** (DMSO-*d*_6_, 45 °C). (A) Fragment of the 1D ^1^H amplitude-modulated spin-echo spectrum measured without (black trace) or with selective inversion of the ^15^N2 (blue trace) or ^15^N3 (red trace) nuclei. The spectrum was measured using a spin-echo delay (delay for the evolution of *J*_HN_) of 1 s. The measured *J*_HN_ values are listed. The signals of ^13^C-DMSO at natural isotope abundance and the signals of impurities are marked by # and *, respectively. The concentration of these impurities relative to the concentration of **15a-^15^N_2_** does not exceed 2%. The up-field region of the spectrum is drawn with increased scaling. (B) Fragment of the 2D ^15^N-HMBC spectrum of **15a-^15^N_2_**. The ^1^H-^15^N cross-peaks between the adamantane protons and ^15^N-labelled atoms are shown.

**^1^H-^15^N couplings for the characterization of N-adamantylation sites in fused azolo-azines.** The signal splittings due to the *J*_HN_ couplings were observed in the 1D ^1^H spectra only in a limited number of cases (compounds **20-^15^N_2_**, **21a,b-^15^N_2_, 23-^15^N_2_** and **24-^15^N_2_**, see Scheme 2 and Scheme 3). In the other cases, the *J*_HN_ couplings were measured by amplitude-modulated 1D ^1^H spin-echo experiments with selective inversion of the ^15^N nuclei [24] (Figure 4A, Table 1) or detected using the conventional 2D ^15^N-HMBC spectra (Figure 4B and Figures S19–S23 in Supporting Information File 1). These methods allowed for the straightforward detection and measurements of the geminal ^2^*J*_HN_ (values of 13.8–16.1 Hz), vicinal ^3^*J*_HN_ (values of 0.83–6.6 Hz) and long-range ^4/5^*J*_HN_ (values of 0.04–0.9 Hz) couplings for the isotopically enriched nitrogen atoms. The ^1^H-^15^N spin–spin interactions with the unlabelled ^15^N nuclei (at natural abundance) were also detected in the ^15^N-HMBC experiments (Table 1). The intensities of the HMBC cross-peaks for the ^15^N-labelled nuclei demonstrated an approximate correlation with the measured *J*_HN_ values (see Table 1). This provides a way to qualitatively estimate the *J*_HN_ magnitudes for unlabelled and ^15^N-labelled nuclei using the relative intensities of the HMBC cross-peaks, corrected for the degree of the isotope enrichment. The measured *J*_HN_ couplings and HMBC ^1^H-^15^N spin–spin interactions are shown in Schemes 1–3.

Compound **15a-^15^N_2_** was characterized by a set of *J*_HN_ couplings detected for the H2' (^3/4^*J*_H2'-N2/N3_ 0.83/0.06 Hz), H3' (^4/5^*J*_H3'-N2/N3_ 0.60/0.11 Hz) and H4' (^5^*J*_H4'-N2_ 0.23 Hz) atoms of the adamantane group (Figure 4A, Table 1, Scheme 1). These spin–spin interactions, with the exception of ^5^*J*_H3'-N3_, were also observed in the 2D ^15^N-HMBC spectrum (Figure 4B). The observed *J*_HN_ pattern indicated that the adamantane substituent is attached to the ^15^N-labelled atom (N2) of the tetrazole ring. Similarly, the observation of the ^5^*J*_H3'-N2_ coupling constant (≈0.04 Hz) and the medium intensity H2'-N1 HMBC cross-peak at natural ^15^N abundance revealed that compound **15b-^15^N_2_** contains an N-adamantane moiety attached to the unlabelled N1 atom (Scheme 1). Note that the weak cross-peak corresponding to the long-range ^5^*J*_H2'-N3_ coupling was observed in the HMBC spectrum of **15b-^15^N_2_** (Figure S20 in Supporting Information File 1), but the magnitude of this *J* coupling was under the limit of reliable *J*_HN_ measurements (0.04 Hz). Thus, if the *J*_HN_ couplings are too small to be measured quantitatively, the ^15^N-HMBC experiment could provide useful information about the position of the adamantane substituent. However, the assignment of the ^15^N-labelled atoms in compounds **15a,b-^15^N_2_** (differentiation between N2 and N3 resonances) could not be achieved using the *J*_HN_ and ^15^N-HMBC data alone. The absence of protons in the tetrazolo[1,5-*b*][1,2,4]triazine core of these compounds dictates the necessity of *J*_CN_ analysis for the unambiguous assignment of ^15^N-labelled nuclei.

In contrast to the situation observed for compounds **15a,b-^15^N_2_**, the *J*_HN_ interactions with the H2 proton in the 1,2,4-triazolo[5,1-*c*][1,2,4]triazines **19-^15^N_2_**, **20-^15^N_2_**, and **21a,b-^15^N_2_** and the H2 and H6 protons in the 1,2,4-triazolo[1,5-*a*]pyrimidines **23-^15^N_2_** and **24-^15^N_2_** permitted the straightforward assignments of the labelled ^15^N atoms (see Scheme 2 and Scheme 3). The attachment of an adamantyl substituent to the N4 atom in compound **21b-^15^N_2_** was confirmed by the measured long-range ^4^*J*_H2'-N5_ coupling constant (0.06 Hz) and the medium intensity H2'-N4 HMBC cross-peak observed at natural ^15^N abundance (Table 1, Scheme 2). Notably, the weak cross-peak corresponding to the ^4^*J*_H2'-N5_ coupling was also detected in the ^15^N-HMBC spectrum (Figure S22D in Supporting Information File 1).

For the adamantylated heterocycles **21a-^15^N_2_** and **24-^15^N_2_**, the *J*_HN_ interactions between the adamantane protons and the labelled N1, N5 or N8 atoms were not detected by amplitude-modulated ^1^H spin-echo or ^15^N-HMBC experiments. Meanwhile, the interactions between the H2' proton of the adamantane and the unlabelled N3 atom of the heterocyclic moieties of the compounds were observed in the ^15^N-HMBC spectra (Scheme 2 and Scheme 3). These results confirmed the coupling of the adamantane bridgehead C1' carbon with the N3 nitrogen of the azole ring in **21a-^15^N_2_** and **24-^15^N_2_**.

The identification of adamantylation sites based on ^15^N-HMBC data requires the preliminary assignment of the nitrogen atoms at natural isotopic abundance. For compounds **21a,b-^15^N_2_** and **24-^15^N_2_,** the required ^15^N assignment could be obtained by observing the ^15^N-HMBC correlations from the H2 and H6 protons. However, the detection of the corresponding cross-peaks was hindered by the presence of large (>3 Hz) *J*_HN_ couplings with the isotopically enriched ^15^N-nuclei. The suppression of the magnetization transfer through the geminal ^2^*J*_H2-N1_ couplings by setting a delay in the ^15^N-HMBC experiment to 1/*J*_HN_ (62.5–71.4 ms) permitted the observation of the correlations between H2 and the unlabelled N3 and N8 atoms in compounds **21a,b-^15^N_2_** (Figures S21 and S22 in Supporting Information File 1). Meanwhile, the presence of additional large vicinal couplings (^3^*J*_H2-N8_ and ^3^*J*_H6-N8_) made this strategy not applicable for compound **24-^15^N_2_**. In this case, the supposed assignment of the N3 resonance was indirectly confirmed by the similarity of its chemical shifts in compounds **21a-^15^N_2_** and **24-^15^N_2_**.

**NMR and Х-ray diffraction data revealing several rotameric configurations of adamantane substituents.** The ^13^C signals of the *N*-adamantyl substituents in compounds **15a,b-^15^N_2_** and **21a,b-^15^N_2_** measured at 45 °C demonstrated additional splitting, which was not connected to the ^1^H-^13^C and ^13^C-^15^N *J*-couplings. The C1' and C2' signals of adamantane (and C3' for **21a-^15^N_2_**) were split into two components with a relative intensity ratio of ≈10:7 and a frequency difference of 0.5–1.2 Hz (Figure 3, Table 2). This revealed the presence of the two structural forms of the N-adamantylated heterocycles in solution with a slow (characteristic time ≥ 1 s) exchange between them. The rotation of the *N*-adamantyl substituents around the N–C1' bond in the bulky bicyclic heterocycles is likely hindered, and the observed conformational heterogeneity corresponds to the different rotameric configurations of the substituent. To test this hypothesis, additional NMR measurements at elevated temperature were carried out for compound **21a-^15^N_2_**. The ^13^C 1D NMR spectrum measured at 70 °C with ^1^H and ^15^N decoupling did not demonstrate additional splitting (Figure S25 in Supporting Information File 1). This confirmed that the studied NMR samples contained unique and chemically pure compounds, while the heterogeneity observed at 45 °C was connected to the presence of different rotameric states.

**Figure 5:**
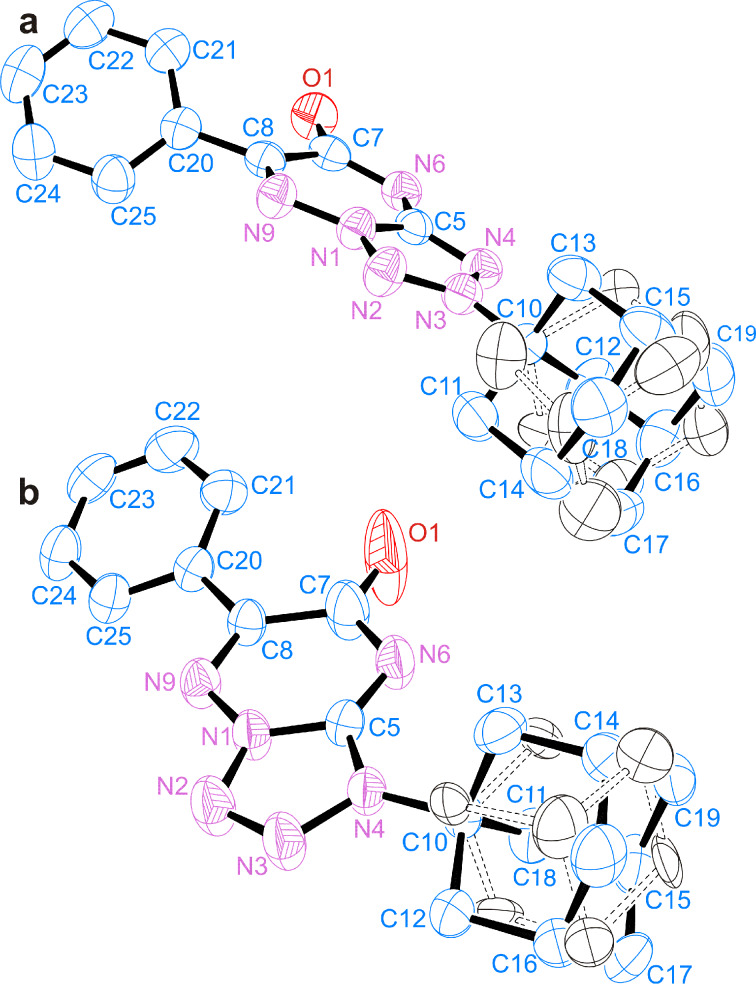
ORTEP diagrams of the X-ray structures of compounds **15a-^15^N_2_** (a) and **15b-^15^N_2_** (b). For clarity, the H atoms are omitted. The observed disorder of the adamantane fragment is shown by black ellipsoids and dashed bonds (the carbon atoms are unlabelled).

To confirm the determined positions of the N-adamantane substitutions, compounds **15a** and **15b** were studied by X-ray crystallography. Suitable crystals of **15a** and **15b** were obtained by slow evaporation from ethyl acetate solutions. The solved X-ray structures were in a full agreement with the results of the *J*_CN_ and *J*_HN_ analysis and confirmed the N2-substituted mesoionic form for compound **15a** as well as the attachment of adamantane to the N1 atom in compound **15b**. In accordance with expectations, the adamantane groups in the crystals of **15a** and **15b** were found disordered between two conformations with different rotameric configurations around the N–C1' bond (Figure 5 and Supporting Information Files 2 and 3). These forms differ by the rotation around the N–C1' bond by 40–60°; thus, in each of them, one of the C2' atoms of the adamantane substituent is located approximately in plane with the heterocyclic moiety of the compound. The populations of the two conformational forms in the single crystals of **15a** and **15b** (4:1 and 17:3, respectively) differ from the populations of the conformers observed by NMR spectroscopy in DMSO solution (≈10:7). Interestingly, for **15a**, the major conformer corresponds to a rotameric state with a screened N1 atom, but in the major conformer of **15b**, the N2 atom of the heterocycle is screened. Notably, similar structural disorder was previously observed in the crystals of adamantylated tetrazolylpyrazole derivatives [37,41].

## Discussion

**Comparison of different NMR approaches for the determination of N-alkylation sites in fused heterocycles.** The obtained data permit a comparison of the abilities of different NMR parameters (^13^C and ^15^N chemical shifts, *J*_HN_ and *J*_CN_) to provide structural information about the N-adamantylation sites in bicyclic heterocycles. The previous studies of azolo[5,1-*c*][1,2,4]triazin-7-ones, 1,2,4-triazolo[1,5-*a*]pyrimidin-7-ones and tetrazolo-azines revealed that the ^13^C chemical shifts of the nearest carbon atoms to N-alkyl fragments could be used as indicators for the formation of N-alkylated azolo-azines [12,42]. For the presently studied compounds, we can expect considerable changes in the chemical shifts of the bridgehead C3a and C8a atoms. The shifts of the other carbon atoms from the heterocyclic parts moieties of the compounds (C2, C5, C6, and C7) may also provide useful structural information.

In the studied tetrazolo-triazines and tetrazolo-pyrimidines (compounds **13-^15^N_2_** and **15a,b-^15^N_2_** and compounds from work [25]), the resonances of the bridgehead C8a atom were observed over a relatively narrow spectral range (144–155 ppm). In the triazolo-triazines and triazolo-pyrimidines **19-^15^N_2_**, **20-^15^N_2_**, **21a,b-^15^N_2_**, **23-^15^N_2_**, and **24-^15^N_2_**, the similar bridgehead C3a atoms are shifted slightly downfield (149–160 ppm). Here, we observed that N-adamantylation of the nitrogen atom directly attached to the C3a, C8a or C2 atoms induced up-field shifts of the corresponding ^13^C signal. The majority of these shifts had relatively small magnitudes (Δδ −0.9 to −2.8 ppm), and a large shift was only observed for the C2 resonance of **21a-^15^N_2_** (Δδ −11.3 ppm (Table 2)). However, these up-field shifts could not be used to determine the N-adamantylation site. The attachment of the adamantane moiety to other nitrogen atoms could lead to similar ^13^C shifts. For example, similar Δδ values (−0.9 and −1.1 ppm) were observed for the C2 resonances in compounds **21b-^15^N_2_** and **24-^15^N_2_**, where the adamantane fragments are attached to N3 and N4, respectively. Note that the C2 and N4 atoms are separated by three covalent bonds.

A similar situation was observed for the carbon atoms that are separated from the N-adamantylation site by two covalent bonds (Table 2). The attachment of adamantane to the N2 atom in compound **15a-^15^N_2_** induced a large down-field shift (Δδ +8.6 ppm) of the C8a resonance, while modification of the N4 atom in compound **21b-^15^N_2_** induced an up-field shift (Δδ −3.0 ppm) of the C6 resonance. Thus, the obtained data did not reveal an easily interpreted correlation between the ^13^C chemical shifts and the position of N-adamantane substituents. The same issue was previously noted in the study of N-alkylated tetrazolo[1,5-*a*]pyridine derivatives [43].

Similar to the situation observed for the ^13^C nuclei, a comparison of the ^15^N chemical shifts in the starting heterocycles **13-^15^N_2_**, **20-^15^N_2_**, and **23-^15^N_2_** and their N-adamantylated derivatives **15a,b-^15^N_2_**, **21a,b-^15^N_2_**, and **24-^15^N_2_** did not reveal a simple correlation with the position of the substituent group (Figure 2, Table 1). Large changes in the ^15^N resonance position were observed for the N2 atom in compounds **15a-^15^N_2_** (Δδ −87.6 ppm) and **15b-^15^N_2_** (Δδ −38.4 ppm) and for the N5 atom in compound **21a-^15^N_2_** (Δδ +54.2 ppm). According to the data reported for tetrazolo[1,5-*a*]pyridines [43], the shielding of the N2 nucleus in compound **15b-^15^N_2_** can be explained by the adamantylation of the neighbouring N1 atom in the tetrazole fragment. In contrast, the coupling of the adamantyl fragment to the N4 atom in compound **21b-^15^N_2_** did not considerable change the chemical shift of the neighbouring ^15^N5 nucleus (Δδ ≈ +2.1 ppm). For clarity, we should mention that the information that could be obtained from the ^15^N chemical shifts is restricted by the pattern of the ^15^N-label incorporation. In some cases, the isotopic labels were located far from the position of the attached adamantane group. This fact could partially explain the lack of correlation between chemical shifts and structure.

The obtained data indicated that changes in the ^13^C and ^15^N chemical shifts could not reliably determine the adamantylation sites in azolo-azines. Therefore, we focused our study on the analysis of ^1^H-^15^N and ^13^C-^15^N spin–spin interactions. Despite the relatively ‘sparse’ placement of ^15^N labels, in all the synthesized compounds, the bridgehead C1' atom of the adamantyl fragment demonstrated detectable *J*_CN_ couplings (Schemes 1–3, Figure 2). The observed *J*_CN_ values greatly varied in magnitude. The direct and vicinal couplings (^1,2^*J*_CN_) were relatively large (6.5–2.7 Hz), while the geminal and long-range interactions (^3,4^*J*_CN_) were small (0.6–0.2 Hz). The fact that the ^1^*J*_CN_ and ^2^*J*_CN_ as well as the ^3^*J*_CN_ and ^4^*J*_CN_ couplings for the C1' atom had similar magnitudes indicated that additional data are required for the unambiguous determination of the adamantylation sites. For this purpose, we measured and analysed the ^13^C-^15^N and ^1^H-^15^N spin–spin interactions for the other atoms of the adamantane groups (Schemes 1–3). These additional sets of ^2-4^*J*_CN_ and ^2-5^*J*_HN_ data reliably identified the N-adamantylation sites in the all studied compounds. The proposed structures of **15a,b-^15^N_2_** were independently confirmed by Х-ray diffraction data.

One of the advantages of *J*_CN_ and *J*_HN_ data compared with chemical shift data is the usefulness of ‘negative’ information. In the majority of the cases, the absence of a detectable ^13^C-^15^N or ^1^H-^15^N spin–spin interaction indicates the remote localization of the adamantane substituent and labelled nitrogen of the heterocycle. The obtained results showed that the structural information provided by the ^1^H-^15^N spin–spin interactions (measured by 1D ^1^H spin-echo experiments or detected in 2D ^15^N-HMBC experiments) is similar to the information obtained from the *J*_CN_ couplings. However, these approaches are not equivalent. On one hand, the acquisition of *J*_HN_ data requires less measurement time and less sophisticated equipment compared with that of *J*_CN_ data (conventional broadband probe and two-channel NMR spectrometer versus triple-resonance probe and three-channel spectrometer, respectively). On the other hand, the structural characterization of the N-adamantylation site(s) in heterocycles based on the *J*_HN_ data requires the preliminary assignment of the ^15^N resonances. Therefore, the combination of these approaches based on the analysis of *J*_CN_ and *J*_HN_ couplings represents the most effective NMR tool for the determination of adamantylation sites in azolo-azines.

**Scheme 5:**
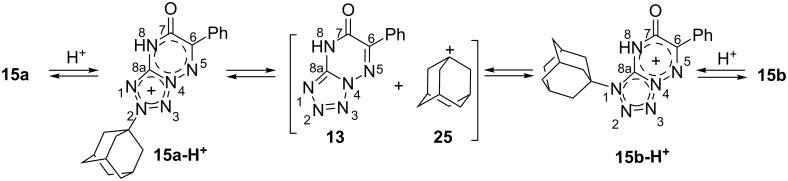
Mechanism of the isomerization of compounds **15a** and **15b**.

**Possible mechanisms of the isomerization of N-adamantylated derivatives 15a and 15b.** The isomerization of unlabelled **15a** in the presence of **13a-^15^N_2_** (Scheme 4) elucidated the possible mechanism of the isomerization of **15a-^15^N_2_** into **15b-^15^N_2_**. This experiment confirmed that this rearrangement occurs via the formation of adamantyl cation **25** and heterocyclic base **13-^15^N_2_** (Scheme 5). Moreover, the equilibration of the isotope composition over the reaction products (15a*-^15^N_2_, 15b*-^15^N_2_ and 13*-^15^N_2_) indicated that the transformation of **15a** into **15b** is reversible. Note that the protonation of compound **13** and its adamantylated derivatives probably plays an important role in the **15a**
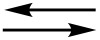
**15b** conversion in TFA solution. The precise positions of the attached protons are unknown, and this determination requires additional investigation, but the analysis of calculated Mulliken charges in compounds **15a** and **15b** (see Supporting Information File 1, Scheme S2) suggests that the most negatively charged atom N8 undergoes the initial protonation. Similar mechanisms can be proposed to describe the isomerization of compounds **21a** and **21b**.

## Conclusion

We reported the selective incorporation of two ^15^N atoms at different positions of 1,2,4-triazolo[1,5-*a*]pyrimidine, azolo-1,2,4-triazines and their N-adamantylated derivatives. The selective incorporation of the ^15^N-labels into the azolo and azine rings of the heterocyclic structures led to the appearance of ^1^H-^15^N and ^13^C-^15^N *J*-coupling constants. The combined analysis of the *J*_HN_ and *J*_CN_ couplings allowed for the effective determination of the adamantylation sites in the azolo-azine series. To the best of our knowledge, the applicability of this approach for the structural determination of N-substituted heterocycles has not been previously considered. We suggest that the proposed method is generally applicable for the studies of N-alkylated heterocyclic compounds with a high abundance of nitrogen nuclei, where ^13^C chemical shifts and ^1^H-^1^H NOE data cannot provide reliable structural information. The incorporation of the ^15^N-labels also permitted the study of the mechanism of isomerization of N-adamantylated tetrazolo[1,5-*b*][1,2,4]triazin-7-one in TFA solution. The formation of an adamantyl cation and NH-tetrazolo-triazine during the isomerization reaction was confirmed.

## Supporting Information

File 1 Detailed experimental procedures, the synthesis of labelled compounds, crystallographic information for **15a-^15^N_2_** and **15b-^15^N_2_**, computational data, and 1D ^1^H, ^13^C and 2D ^15^N-HMBC spectra of the synthesized compounds.

File 2 Crystallographic data for **15a-^15^N_2_**.

File 3 Crystallographic data for **15b-^15^N_2_**.
