# Supplementary material for: 15N-Labelling and structure determination of adamantylated azolo-azines in solution
Source: Beilstein J Org Chem. 2017 Nov 29;13:2535–48. doi: 10.3762/bjoc.13.250 (PMC5727827; doi:10.3762/bjoc.13.250)
Supplement: File 1 — Detailed experimental procedures, the synthesis of labelled compounds, crystallographic information for 15a-15N2 and 15b-15N2, computational data, and 1D 1H, 13C and 2D 15N-HMBC spectra of the synthesized compounds. [file Beilstein_J_Org_Chem-13-2535-s001.pdf]

Supporting Information File 1
for

^{15}N-Labelling and structure determination of adamantylated azolo-azines in solution

Sergey L. Deev[1*], Alexander S. Paramonov[2], Tatyana S. Shestakova[1], Igor A. Khalymbadzha[1], Oleg N. Chupakhin[1,3], Julia O. Subbotina[1], Oleg S. Eltsov[1], Pavel A. Slepukhin[1,3], Vladimir L. Rusinov[1], Alexander S. Arseniev[2] and Zakhar O. Shenkarev[2]

Address: [1]Ural Federal University, 19 Mira Street, 620002 Yekaterinburg, Russia, [2]Shemyakin-Ovchinnikov Institute of Bioorganic Chemistry, Russian Academy of Sciences, 16/10 Miklukho-Maklaya Street, 117997 Moscow, Russia and [3]I. Ya. Postovsky Institute of Organic Synthesis, Ural Branch of the Russian Academy of Sciences, 22 S. Kovalevskoy Street, 620219 Yekaterinburg, Russia

Email: Sergey L. Deev - deevsl@yandex.ru

* Corresponding author

Table of Contents

General methods and procedures S2

X-ray crystallographic analysis S4

Synthesis and experimental procedures S5

Quantum chemical calculations S9

References S15

NMR spectra S16

General methods and procedures

Instrumentation and measurements. Melting points were determined on a Boetius melting-point apparatus and are uncorrected. High-resolution mass spectrometry (HRMS) and Fourier transform mass spectrometry (FTMS) were carried out with electrospray ionization (ESI) using a Bruker maXis UHR-TOF mass spectrometer. IR spectra were recorded using a Bruker Alpha FTIR spectrometer equipped with an ATR sampling accessory; absorbance frequencies are given at maximum intensity in cm^{-1}. All NMR data for the synthesized $^{15}N_2$-compounds were obtained in DMSO-d_6 solution at 45 °C. NMR spectra were recorded using samples with concentrations in the range of 30–70 mM. The 1D ^{1}H, ^{13}C and ^{15}N NMR spectra and 2D ^{13}C-HMQC and ^{13}C-HMBC experiments were acquired using a Bruker AVANCE-700 spectrometer equipped with a triple-resonance (^{1}H, ^{13}C, ^{15}N) room-temperature probe. The operating frequencies for ^{1}H, ^{13}C and ^{15}N were 700 MHz, 175 and 71 MHz, respectively. The 2D ^{15}N-HMBC spectra were acquired on a Bruker AVANCE-400 spectrometer (^{1}H 400 MHz, ^{15}N 41 MHz). The 2D ^{13}C-HMQC, ^{13}C-HMBC and ^{15}N-HMBC spectra were measured using standard Bruker pulse sequences with gradient selection of the magnetization transfer pathway. A low-pass *J*-filter was used in the ^{13}C-HMBC experiments to suppress one-bond correlations. The delay for the long-range magnetization transfer in the ^{13}C-HMBC experiments was set to 125 ms (corresponding to a J_{HC} value of 4 Hz). Delays of 62.5-125 ms were used in the ^{15}N-HMBC experiments (corresponding to J_{HN} values of 4-8 Hz). The ^{1}H chemical shifts were referenced to the residual CD_2H signal of DMSO-d_6 at 2.50 ppm. The ^{13}C and ^{15}N chemical shifts were indirectly referenced to TMS and $MeNO_2$, respectively [S1]. Using this indirect referencing scale, the ^{13}C signal of DMSO-*d*$_6$ was observed at 40.155 ppm.

The J_{HN} couplings were detected and measured using an amplitude-modulated spin-echo technique [S2-S4]. The pulse sequence published in [S3] was used. The *J* coupling values were quantitatively extracted from the peak intensities of the multiplets in the 1D ^{1}H spin-echo spectra, which were measured in an interleaved fashion with or without the selective inversion of the ^{15}N nucleus of interest, in the middle of the echo period. The 1D ^{1}H spin-echo spectra were measured with a digital resolution of 1.2 Hz per real point and with spectral width of 20 ppm using a Bruker AVANCE-700 NMR spectrometer. For the reference experiments, the inversion pulse was applied outside the studied spectral region. B0 *z*-gradients were used to remove any imperfections that were associated with the ^{1}H-refocusing and ^{15}N inversion pulses in the middle of the spin-echo. The antiphase $^{1}H_X{}^{15}N_Z$ magnetization was suppressed by broadband ^{15}N decoupling during acquisition [S4]. The spin-echo delays $2 \times \Delta$ (the delay for the evolution of J_{HN}) were set from 0.4 to 1.0 s. The transverse relaxation rates (R_2) for a given ^{1}H nucleus were

assumed to be identical among the ^{15}N-labelled and unlabelled variants of the compound. The following equation was used to extract the *J* couplings:

$$J_{HN} = ArcCos\left[\frac{I^C/I^D-(1-a_i)}{a_i}\right]\Big/2\pi\Delta \qquad (1),$$

where I^C (coupled) and I^D (decoupled) are the intensities of the ^{1}H multiplets measured *with* and *without* the selective inversion of the ^{15}N nucleus, respectively, and a_i is the degree of ^{15}N enrichment (0.98).

The J_{CN} couplings were measured by line-shape analysis in the 1D ^{13}C spectra acquired in an interleaved fashion with broadband decoupling from ^{1}H nuclei and with or without band-selective decoupling of the ^{15}N nucleus of interest [S3]. For the reference experiments, the ^{15}N decoupling was applied outside the studied spectral region. The 1D ^{13}C spectra were measured with a digital resolution of 0.18 Hz per real point and with a spectral width of 170 ppm using a Bruker AVANCE-700 NMR spectrometer. Before analysis, the spectra were processed to a digital resolution of ≈0.06 Hz per complex point using a zero-filling and weak exponential weighting function (LB=0.1). The line shapes of the multiplets in the 1D ^{13}C spectra were fitted to the sum of several Lorentz lines using the 'NonlinearModelFit' function of the Mathematica program (version 7.0, Wolfram research).

Band-selective inversion and decoupling of the ^{15}N nuclei were achieved using WURST-20 adiabatic pulses [S5] with a length of 20 ms and an inversion range of approximately 1 kHz (14 ppm on ^{15}N). For compound **15b-^{15}N$_2$**, 40 ms WURST-20 pulses with an inversion range of 500 Hz were used. All the used NMR pulse sequences and parameter sets are available from the authors upon request.

General Methods. Na^{15}NO$_2$ (98%, ^{15}N), [^{15}N$_2$]-hydrazine sulphate (98%, ^{15}N) and the reagents for which the preparation is not described were obtained from commercial suppliers and used without further purification.

X-ray crystallographic analysis.

The X-ray diffraction data of compounds **15a-$^{15}N_2$** and **15b-$^{15}N_2$** were collected on a Xcalibur S diffractometer with Mo Kα radiation (λ = 0.71073 Å, graphite monochromator, ω/2θ-scanning technique). Unit cell parameters were refined using all collected spots after the integration process. The structures were solved by direct methods that were implemented in the SHELXS-97 program [**S6**]. The refinements were carried out through full matrix anisotropic least-squares methods on F^2 for all reflections of the non-H atoms using the SHELXL-97 program [**S7**]. The crystal data and data collection parameters are summarized in Table S1. The details of the refinement and the final R indices are also presented in Table S1. The X-ray CIF files have been deposited at the Cambridge Crystallographic Data Centre, allocated with the deposition numbers CCDC 1492968 (**15a-$^{15}N_2$**) and CCDC 1493023 (**15b-$^{15}N_2$**). Copies of the data can be obtained, free of charge, from the CCDC, 12 Union Road, Cambridge, CB2 1EZ U.K. (www.ccdc.cam.ac.uk).

Table S1. Crystal data and structure refinement for compounds **15a-$^{15}N_2$** and **15b-$^{15}N_2$**

**Compound**	**15a-$^{15}N_2$**	**15b-$^{15}N_2$**
Crystal size, mm	0.25×0.20×0.15	0.25×0.20×0.15
Crystal colour	colourless	colourless
Empirical formula	$C_{19}H_{20}N_6O$	$C_{19}H_{20}N_6O$
Crystal system	Triclinic	orthorhombic
Space group	P-1	$P2_1/2_1/2_1$
a, Å	6.6443(9)	6.3122(6)
b, Å	9.7386(14)	14.6969(16)
c, Å	13.869(2)	17.880(2)
α	96.807(12)	90
β	101.217(12)	90
γ	99.915(12)	90
Volume ($Å^3$), *Z*	856.3(2), 2	1658.7(3), 4
μ, mm^{-1}	0.089	0.092
Reflections collected	4922	6610
Independent reflections(R_{int})	4069 (0.0140)	3746 (0.0308)
S	1.025	1.074
R_1 $[I>2\sigma(I)]$	0.0373	0.0413
wR_2 $[I>2\sigma(I)]$	0.0960	0.0570
R_1 (all data)	0.0703	0.0913
wR_2 (all data)	0.1027	0.0613
Largest diff. peak and hole, ē/$Å^3$	0.132/-0.129	0.202/-0.186
Completeness to θ (deg.)	96.0% (28.28)	97.5% (28.28)

Synthesis and experimental procedures

Compounds **7-$^{15}N_2$** [**S8**], **9** [**S9**] and **16-^{15}N** [**S10**] were prepared as previously described.

Synthesis of [1,2-$^{15}N_2$]-5-Amino-1,2,4-triazole 16-$^{15}N_2$. The preparation of amino-1,2,4-triazole **16-$^{15}N_2$** was based on the Maquestiau method [S11], which included the interaction of [$^{15}N_2$]-aminoguanidine carbonate with formic acid. In turn [$^{15}N_2$]-aminoguanidine was obtained by the reaction of $^{15}N_2$-hydrazine sulphate and S-methylisothiourea sulphate (Scheme S1).

Scheme S1. Synthesis of [1,2-$^{15}N_2$]-5-amino-1,2,4-triazole 16-$^{15}N_2$

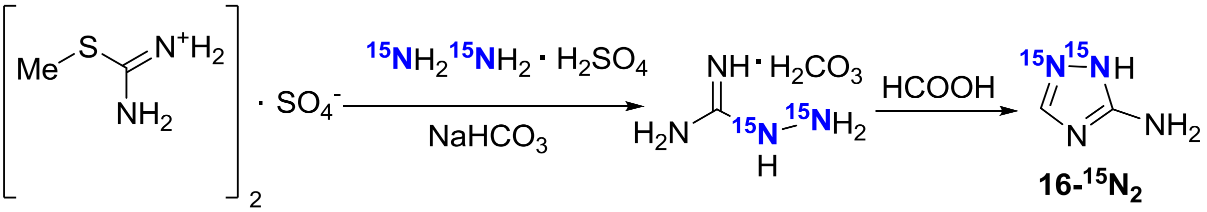

16-$^{15}N_2$

[$^{15}N_2$]-Aminoguanidine carbonate. To a solution of KOH (850 mg, 15.2 mmol) in water (15 mL) [$^{15}N_2$]-hydrazine sulfate (1070 mg, 8.1 mmol), and *S*-methylisothiourea sulfate (1143 mg, 4.57 mmol) were successively added at ambient temperature. The reaction mixture was stirred for 24 h. with air bubbling. Then $NaHCO_3$ (2041 mg, 24.3 mmol) was added, and the mixture was immediately stirred. After ca. 5 min, [$^{15}N_2$]-aminoguanidine carbonate precipitated as white crystals. The precipitate was filtered, washed with ice-cold water, and dried in air. Yield 79% (883 mg, 6.40 mmol).

[1,2-$^{15}N_2$]-5-Amino-1,2,4-triazole 16-$^{15}N_2$. To a sample of $^{15}N_2$-aminoguanidine bicarbonate (1380 mg, 10 mmol) formic acid (0.37 mL, 460 mg, 10 mmol), was carefully added, and reaction mixture was heated at 120 °C for 4 h. After cooling, the mixture was treated with hot iPrOH (ca. 15 mL), and precipitate (if required) was filtered off. The filtrate was evaporated yielding crude [1,2-$^{15}N_2$]-5-amino-1,2,4-triazole (860 mg, 10 mmol, 100%) as a white solid. Compound **16-$^{15}N_2$** was used without further purification.

Synthesis of [2,3-$^{15}N_2$]-tetrazolyldiazonium salt (8-$^{15}N_2$): CAUTION! Tetrazolyldiazonium salts are very sensitive to electrostatic discharge, friction, and impact. During this work, the following safety precautions were obeyed: protective clothing, a face shield and ear plugs were utilized. Concentrated HCl (0.5 mL) was added to a solution of [2,3-$^{15}N_2$]-aminotetrazole hydrate **7-$^{15}N_2$** (200 mg, 1.90 mmol) in water (10 mL). After cooling the reaction mixture to -2 °C, a solution of $NaNO_2$ (144.9 mg, 2.1 mmol) in water (2 mL) was added dropwise under

vigorous stirring. The reaction mixture was kept at −2 °C for 30 min. The resulting solution of the diazonium salt (**8-^{15}N$_2$**) was used for the synthesis of **13-^{15}N$_2$**.

Synthesis of [2,3-^{15}N$_2$]-6-phenyltetrazolo[1,5-*b*][1,2,4]triazin-7-one (13-^{15}N$_2$): A mixture of ethyl phenyl(formyl)acetate (**9,** 400 mg, 2.08 mmol) and sodium carbonate (600 mg) in water (3 mL) and ethanol (2 mL) was added to the resulting solution of diazonium salt **8-^{15}N$_2$**. The resulting solution was stirred at room temperature for 2 h and then concentrated HCl (1 mL) was added. The precipitate was filtered off and then dissolved in 2 mL of acetic acid. The solution was refluxed for 2 h and then cooled. The precipitate formed was filtered off and dried to give compound **13-^{15}N$_2$** (226 mg, 55%); mp 225 °C; IR (Zn−Se ATR, cm^{-1}) 2942, 2852, 1716, 1634, 1538, 956, 753, 699; HRMS (ESI) calcd. for $C_9H_7N_4{}^{15}N_2O$ [M+H]$^+$, 217.0622; found, 217.0617.

Adamantylation of [2,3-^{15}N$_2$]-tetrazolo[1,5-*b*][1,2,4]triazin-7-one (13-^{15}N$_2$): Procedure A. 1-Adamantanol (**14**, 106 mg, 0.697 mmol) was added to a solution of compound **13-^{15}N$_2$** (100 mg, 0.463 mmol) in TFA (2 mL). The reaction mixture was refluxed for 5 min. After cooling the solution to room temperature, the acid was removed in vacuo. The residue was recrystallized from 2-propanol. Yield **15a-^{15}N$_2$** 45 mg (28%) of colourless crystals; mp 312 °C; IR (Zn−Se ATR, cm^{-1}) 2905, 1646, 1575, 1523, 692; HRMS for $C_{19}H_{21}N_4{}^{15}N_2O$ [M+H]$^+$: calcd, 351.1718, found, 351.1712.

Procedure B. 1-Adamantanol (**14,** 106 mg, 0.697 mmol) was added to a solution of compound **13-^{15}N$_2$** (100 mg, 0.463 mmol) in TFA (2 mL). The reaction mixture was refluxed for 2 h. After cooling the solution to room temperature, the acid was removed in vacuo. The residue was purified by column chromatography (hexane/EtOAc 5:1→1:10) on silica gel to afford compounds **15a-^{15}N$_2$** (25 mg, 15%) and **15b-^{15}N$_2$** (65 mg, 40%) as colourless crystals; mp 246 °C; IR (Zn−Se ATR, cm^{-1}) 2856, 1668, 1580, 1505, 1487, 1434, 841, 688; HRMS for $C_{19}H_{21}N_4{}^{15}N_2O$ [M+H]$^+$: calcd, 351.1718, found, 351.1712.

Isomerization of 15a-^{15}N$_2$ to 15b-^{15}N$_2$ in the presence of 1-adamantanol: 1-Adamantanol (**14**, 106 mg, 0.697 mmol) was added to a solution of compound **15a-^{15}N$_2$** (100 mg, 0.463 mmol) in TFA (3 mL). The reaction mixture was refluxed for 2 h. After cooling the solution to room temperature, the acid was removed in vacuo. The residue was purified by column chromatography (hexane/EtOAc 5:1→1:10) on silica gel to afford compounds **15a-^{15}N$_2$** (21 mg, 13%) and **15a-^{15}N$_2$** (57 mg 35%).

Synthesis of the sodium salt of [1,5-^{15}N$_2$]-6-nitro-1,2,4-triazolo[5,1-*c*][1,2,4]triazin-7-one (19-^{15}N$_2$): Concentrated 70% HNO_3 (0.5 mL) was added to a solution of 5-amino-1,2,4-triazole **16-^{15}N** (174 mg, 2.02 mmol) in water (1.5 mL). The reaction mixture was cooled to 0 °C, and a solution of $Na^{15}NO_2$ (150 mg, 2.14 mmol) in water (1 mL) was added dropwise under vigorous

stirring. The reaction mixture was kept at 0 °C for 10 min and then added to a mixture of ethyl nitroacetate **18** (269 mg, 0.220 mL, 2.02 mmol) and a 17% water solution of Na_2CO_3 (7 mL) at 0 °C. The reaction mixture was stirred at room temperature for 2 h. The precipitate was filtered off and recrystallized from 50% acetic acid to give the titled compound. Yield **19-^{15}N$_2$**152 mg (31%).

Synthesis of [1,5-^{15}N$_2$]-6-Bromo-1,2,4-triazolo[5,1-*c*][1,2,4]triazin-7-one (20-^{15}N$_2$): Acetyl bromide (2 mL, 27.05 mmol) was added dropwise over 10 min into a mixture of the sodium salt of 6-nitro-1,2,4-triazolo[5,1-*c*][1,2,4]triazin-7-one dihydrate **19-^{15}N$_2$** (300 mg, 1.239 mmol) and ethanol (2 mL). The reaction mixture was refluxed until the evolution of nitrous gases ceased (15 min). After cooling the solution to room temperature, ethanol (5 mL) was added. The precipitate formed was filtered off and dried to give compound **20-^{15}N$_2$**, which was used without further purification. Yield 131 mg (49%); mp 315 °C; IR (Zn−Se ATR, cm^{-1}) 2832, 1723, 1608, 1496, 1162, 740; HRMS for $C_4H_3N_3{}^{15}N_2OBr$ $[M+H]^+$: calcd, 217.9462, found, 217.9453.

Adamantylation of [1,5-^{15}N$_2$]-1,2,4-triazolo[5,1-*c*][1,2,4]triazin-7-one (20-^{15}N$_2$): 1-Adamantanol (**14,** 106 mg, 0.697 mmol) was added to a solution of compound **20-^{15}N$_2$** (100 mg, 0.458 mmol) in TFA (2 mL). The reaction mixture was refluxed for 10 min. After cooling the solution to room temperature, the acid was removed in vacuo. The residue was recrystallized from 2-propanol. Yield **21a-^{15}N$_2$** 45 mg (28%) of colourless crystals; mp 339 °C; IR (Zn−Se ATR, cm^{-1}) 2912, 1708, 1549, 15,14, 1036, 747; HRMS for $C_{14}H_{17}N_3{}^{15}N_2OBr$ $[M+H]^+$: calcd, 352.0557, found, 352.0553.

Isomerization of 21a-^{15}N$_2$ to 21b-^{15}N$_2$ in the presence of 1-adamantanol: 1-Adamantanol **14** (35 mg, 0.230 mmol) was added to a solution of compound **21a-^{15}N$_2$** (53 mg, 0.151 mmol) in TFA (2 mL). The reaction mixture was refluxed for 6 h. After cooling the solution to room temperature, the acid was removed in vacuo. The residue was recrystallized from 2-propanol. Yield **21b-^{15}N$_2$** 23 mg (43%) of colourless crystals; mp 291 °C; IR (Zn−Se ATR, cm^{-1}) 2902, 2854, 1729, 1546, 1166, 739; HRMS for $C_{14}H_{17}N_3{}^{15}N_2OBr$ $[M+H]^+$: calcd, 352.0557, found, 352.0552.

Synthesis of [1,8-^{15}N$_2$]-5-trifluoromethyl-1,2,4-triazolo[1,5-*a*]pyrimidin-7-one (23-^{15}N$_2$): Ethyl 4,4,4-trifluoroacetoacetate (**22**, 0.60 mL, 4.103 mmol) was added to a solution of [1,2-^{15}N$_2$]-amino-1,2,4-triazole **16-^{15}N$_2$** (300 mg, 3.488 mmol) in 3 mL of acetic acid. The reaction mixture was refluxed for 2 h. After cooling the solution to room temperature, the precipitate was filtered off and dissolved in 2 mL of concentrated H_2SO_4. Then, 8 g of ice was added to the obtained solution, and the precipitate was filtered off, washed with cold water and dried to afford compound **23-^{15}N$_2$** (310 mg, 43%); mp 241 °C; IR (Zn−Se ATR, cm^{-1}) 3108, 1682, 1635, 1136, 843, 758; HRMS for $C_6H_4N_2{}^{15}N_2OF_3$ $[M+H]^+$: calcd, 207.1042, found, 207.0276.

Adamantylation of [1,8-$^{15}N_2$]-5-trifluoro-1,2,4-triazolo[1,5-*a*]pyrimidin-7-one (23-$^{15}N_2$): 1-Adamantanol (**14,** 70 mg, 0.460 mmol) was added to a solution of compound **23-$^{15}N_2$** (60 mg, 0.291 mmol) in TFA (3 mL). The reaction mixture was refluxed for 8 h. After cooling the solution to room temperature, the acid was removed in vacuo. The residue was recrystallized from 2-propanol. Yield **24-$^{15}N_2$** 40 mg (40%) of colourless crystals; mp 229 °C; IR (Zn−Se ATR, cm^{-1}) 2916, 1698, 1539, 1135, 923; HRMS for $C_{16}H_{18}N_2{}^{15}N_2OF_3$ $[M+H]^+$: calcd, 341.1373, found, 341.1367.

Quantum chemical calculations

The DFT calculations were carried out using the Gaussian 09 program [**S12**]. All structures were optimized with the RB3LYP/6-31-G(d,p) method in the gas phase. The formation energies of compounds **15a-$^{15}N_2$** (E_1) relative to **15b-$^{15}N_2$** (E_2) and **21a-$^{15}N_2$** (E_1) relative to **21b-$^{15}N_2$** (E_2) were calculated. The unit of the energy was 1 Hartree or 1 au of energy (0.01Hartree = 6.2752 kcal/mol). Analysis of the relative formation enthalpies ($\Delta E = E_1 – E_2$) showed that isomers **15b-$^{15}N_2$/21b-$^{15}N_2$** are more thermodynamically stabile than compounds **15a-$^{15}N_2$/21a-$^{15}N_2$**. (ΔE = 0.013266/0.010125 Hartree or 8.3/6.4 kcal/mol).

Data 1: Cartesian coordinates and energy of **15a-$^{15}N_2$**

	X	Y	Z
**N**	**0.8385480727**	**-0.2647576064**	**-1.777456223**
**C**	**-0.2336352557**	**-0.3660520446**	**-1.0384398002**
**N**	**-0.3229290804**	**0.118861633**	**0.2605776398**
**N**	**0.6598239088**	**0.7502954446**	**0.9272187687**
**C**	**1.7713448144**	**0.8744753232**	**0.2392539238**
**C**	**1.9211534284**	**0.3703354415**	**-1.2022466441**
**O**	**2.9751695787**	**0.5281087009**	**-1.8073708593**
**C**	**2.8859696557**	**1.5216355912**	**0.9668458758**
**N**	**-1.4419375101**	**-0.9223141434**	**-1.311323886**
**N**	**-2.1325724209**	**-0.7286591566**	**-0.1885457894**
**N**	**-1.5275945792**	**-0.1124479514**	**0.7926753754**
**C**	**3.9743956816**	**2.1195087696**	**0.3044216938**
**C**	**4.9819314858**	**2.7460238915**	**1.0372720931**
**C**	**4.9314198538**	**2.7807759268**	**2.4299256418**
**C**	**3.8569264433**	**2.1856230893**	**3.0978049812**
**C**	**2.8446885923**	**1.5657776219**	**2.376179074**
**C**	**-4.3851786113**	**-0.5675195834**	**-1.1749081709**
**C**	**-5.8297809227**	**-1.1029351134**	**-1.0778016057**
**C**	**-6.4153440217**	**-0.7497101715**	**0.3043198836**
**C**	**-5.5538446491**	**-1.3989044207**	**1.4066683293**
**C**	**-4.1065075753**	**-0.8640957861**	**1.3193382703**
**C**	**-3.5339492687**	**-1.2226241943**	**-0.0638384027**
**C**	**-5.8236119124**	**-2.6346033577**	**-1.262742783**
**C**	**-4.9673793067**	**-3.2840663542**	**-0.1559994268**
**C**	**-3.520487558**	**-2.7571217583**	**-0.2492120108**
**C**	**-5.5531204351**	**-2.9309104744**	**1.2262606238**
**H**	**4.0223226591**	**2.0765226956**	**-0.7738566242**
**H**	**5.8130399165**	**3.2061364351**	**0.5111677991**
**H**	**5.7242941386**	**3.2633472812**	**2.9942965701**
**H**	**3.8123346295**	**2.2013543189**	**4.1828084395**
**H**	**2.0124099438**	**1.1027051004**	**2.8935801898**
**H**	**-4.3691113568**	**0.5220362027**	**-1.0506749888**
**H**	**-3.9469943692**	**-0.7951058156**	**-2.1511568752**
**H**	**-6.4293477785**	**-0.6356888502**	**-1.8676325652**
**H**	**-7.4496542583**	**-1.1074906711**	**0.3779560416**
**H**	**-6.4441470133**	**0.3392306973**	**0.4371821964**
**H**	**-5.9573833045**	**-1.1416361958**	**2.3927721681**
**H**	**-3.4877501909**	**-1.3038770543**	**2.1084450823**
**H**	**-4.0886340705**	**0.2213248316**	**1.4636189978**
**H**	**-5.4256070133**	**-2.8949381725**	**-2.251425931**
**H**	**-6.8489637445**	**-3.0216542017**	**-1.218670115**
**H**	**-4.9526811665**	**-4.3722404468**	**-0.2877811648**
**H**	**-2.8936027567**	**-3.2114311642**	**0.5276186383**
**H**	**-3.0751740548**	**-3.0018264918**	**-1.2181995458**
**H**	**-6.5754999645**	**-3.318639916**	**1.31250155**
**H**	**-4.9633486536**	**-3.4058279002**	**2.0205735644**

(E_1) Sum of electronic and zero-point Energies = -1139.385874 Hartree

Data 2: Cartesian coordinates and energy of **15b-$^{15}N_2$**

	X	Y	Z
**N**	**-3.4574895887**	**-0.6844541398**	**-0.1652379646**
**C**	**-2.2258968981**	**-0.5944999063**	**0.2494810042**
**N**	**-1.5913936689**	**0.5603567372**	**0.6018304061**
**N**	**-2.1526159435**	**1.7827510626**	**0.5596366285**
**C**	**-3.3939725881**	**1.7948686999**	**0.1395433809**
**C**	**-4.1505355394**	**0.5198171466**	**-0.2402057515**
**O**	**-5.3174289384**	**0.5572718209**	**-0.6025593082**
**C**	**-4.0127205869**	**3.1358018001**	**0.0306411701**
**N**	**-1.2597481101**	**-1.5406620135**	**0.449084195**
**N**	**-0.1218176947**	**-0.9336330696**	**0.9040925531**
**N**	**-0.3063642942**	**0.3258422947**	**1.0004437288**
**C**	**-5.4061755605**	**3.3294695127**	**0.0359316806**
**C**	**-5.933997753**	**4.618431441**	**-0.0336401603**
**C**	**-5.0931240069**	**5.7271084732**	**-0.1179519437**
**C**	**-3.7072180012**	**5.5444422539**	**-0.1285242127**
**C**	**-3.1719880009**	**4.2646236615**	**-0.0529251117**
**C**	**-2.4436410367**	**-3.5942609217**	**1.1571041413**
**C**	**-2.546509536**	**-5.1163981258**	**0.9211509449**
**C**	**-1.1897602899**	**-5.7777804504**	**1.2367049126**
**C**	**-0.1050872752**	**-5.1878744026**	**0.3128276309**
**C**	**0.0056711956**	**-3.6656896693**	**0.5478867142**
**C**	**-1.3524521417**	**-3.0098594019**	**0.2294023545**
**C**	**-2.9222677587**	**-5.3821836108**	**-0.5515021937**
**C**	**-1.8323667855**	**-4.8001385312**	**-1.4753416507**
**C**	**-1.72516144**	**-3.2773709985**	**-1.248113848**
**C**	**-0.4764553083**	**-5.4618994872**	**-1.1588955224**
**H**	**-6.0606700528**	**2.4714786495**	**0.0852193223**
**H**	**-7.0115883355**	**4.7521665156**	**-0.0243507749**
**H**	**-5.5116367741**	**6.7275829277**	**-0.1794954245**
**H**	**-3.043927891**	**6.4012526809**	**-0.2007763007**
**H**	**-2.0975610474**	**4.1215867888**	**-0.0623708461**
**H**	**-2.1810608587**	**-3.3840783407**	**2.201352886**
**H**	**-3.4010821409**	**-3.1063071609**	**0.9486209351**
**H**	**-3.3221615842**	**-5.5229092498**	**1.580851028**
**H**	**-1.2563991639**	**-6.8631876364**	**1.0911657967**
**H**	**-0.9227986647**	**-5.6124901032**	**2.2883973184**
**H**	**0.8648683653**	**-5.6448633536**	**0.5418406102**
**H**	**0.7873152705**	**-3.2352159531**	**-0.0871648254**
**H**	**0.2878963773**	**-3.4584360917**	**1.5854502829**
**H**	**-3.8939222334**	**-4.9283144815**	**-0.7815287362**
**H**	**-3.0220162878**	**-6.4610602462**	**-0.7238537123**
**H**	**-2.0997585338**	**-4.9807978982**	**-2.5231529765**
**H**	**-0.9545846388**	**-2.8454095854**	**-1.8987191427**
**H**	**-2.6738517033**	**-2.7848655471**	**-1.4819990101**
**H**	**-0.5328371468**	**-6.5431426224**	**-1.3361473554**
**H**	**0.3022945939**	**-5.0690694681**	**-1.8252028529**

(E_2) Sum of electronic and zero-point Energies = -1139.399140 Hartree

Data 3: Cartesian coordinates and energy of **21a-$^{15}N_2$**

	X	Y	Z
**C**	**0.6696122564**	**-0.8135547472**	**-1.3965938573**
**C**	**1.4999544116**	**-0.127452301**	**-0.2911551831**
**C**	**1.1862163572**	**-0.7831870474**	**1.0683707576**
**C**	**-0.3147576103**	**-0.6189524736**	**1.3798651003**
**C**	**-1.1502757351**	**-1.302904208**	**0.274843816**
**C**	**-0.8363873461**	**-0.6466783908**	**-1.0855171621**
**C**	**1.1440886827**	**1.3727545115**	**-0.2372978879**
**C**	**-0.3562627474**	**1.5358531013**	**0.0828819688**
**C**	**-1.1944267574**	**0.8567449494**	**-1.0209689704**
**C**	**-0.6698858269**	**0.8800207013**	**1.4423834194**
**N**	**-2.5178458671**	**-1.848995035**	**-4.0818751382**
**N**	**-3.0957819578**	**-2.7005345814**	**-3.1651875961**
**C**	**-2.5455619995**	**-2.3396422732**	**-2.0407826013**
**N**	**-1.6431216927**	**-1.3061021594**	**-2.1566337048**
**C**	**-1.6265677633**	**-0.985672476**	**-3.4979195792**
**N**	**-0.9222369096**	**-0.0610262995**	**-4.1126483277**
**N**	**-1.1252368654**	**0.0156017651**	**-5.4589840588**
**C**	**-1.9543843859**	**-0.7716475039**	**-6.0724303925**
**C**	**-2.7853910833**	**-1.8339735895**	**-5.4744460945**
**O**	**-3.5670043523**	**-2.5858281329**	**-6.0092868929**
**Br**	**-2.1469511787**	**-0.5634599353**	**-7.952833122**
**H**	**0.9117994476**	**-1.8825142354**	**-1.4514151289**
**H**	**0.8882477383**	**-0.3725265896**	**-2.3731627164**
**H**	**2.5639435663**	**-0.2480802936**	**-0.5257452424**
**H**	**1.7842637188**	**-0.3174843002**	**1.8612375709**
**H**	**1.4556772835**	**-1.8471035667**	**1.0486365432**
**H**	**-0.5504040791**	**-1.0973382106**	**2.3377771329**
**H**	**-2.2184389731**	**-1.2030509907**	**0.5065209493**
**H**	**-0.9118813543**	**-2.3737835686**	**0.2432272488**
**H**	**1.3794167481**	**1.8508908903**	**-1.195661356**
**H**	**1.7476257762**	**1.8738520866**	**0.5295425158**
**H**	**-0.6150991046**	**2.6005769339**	**0.1148571448**
**H**	**-2.2661169439**	**0.9651088003**	**-0.8110430815**
**H**	**-0.9977154688**	**1.3174512714**	**-1.9931068947**
**H**	**-0.0945993244**	**1.3661257374**	**2.2398324973**
**H**	**-1.731318971**	**1.0086804507**	**1.690821509**
**H**	**-2.7781916886**	**-2.8111682898**	**-1.1001031855**

(E_1) Sum of electronic and zero-point Energies = -3463.504994 Hartree

Data 4: Cartesian coordinates and energy of **21b-$^{15}N_2$**

	X	Y	Z
**C**	**0.7173279615**	**-0.8126723328**	**-1.3709150856**
**C**	**1.4870489379**	**-0.052989317**	**-0.2690804939**
**C**	**1.1983542016**	**-0.6986603528**	**1.0998603098**
**C**	**-0.3148607012**	**-0.6255191396**	**1.3850477264**
**C**	**-1.0912722791**	**-1.3823357841**	**0.2848851348**
**C**	**-0.8007396143**	**-0.73224606**	**-1.0829534532**
**C**	**1.0350453471**	**1.4222200774**	**-0.251922777**
**C**	**-0.4762146414**	**1.4966326415**	**0.0504068357**
**C**	**-1.2570979367**	**0.7457665309**	**-1.0496236351**
**C**	**-0.7626771095**	**0.8492075745**	**1.4189915851**
**N**	**-2.212613133**	**-1.8337702747**	**-4.4140000182**
**N**	**-1.9993263205**	**-1.307315062**	**-5.6570933208**
**C**	**-1.1713897589**	**-0.3137734071**	**-5.3881532047**
**N**	**-0.8211871353**	**-0.1405468618**	**-4.0781949566**
**C**	**-1.4922704005**	**-1.1128481886**	**-3.4856395533**
**N**	**-1.5574382462**	**-1.468159421**	**-2.1636042455**
**N**	**-2.3195438582**	**-2.5092548728**	**-1.7980423755**
**C**	**-2.9937828133**	**-3.1839957835**	**-2.668693579**
**C**	**-3.0403006171**	**-2.9422051328**	**-4.1262720994**
**O**	**-3.6613210477**	**-3.5572562474**	**-4.9588444505**
**Br**	**-4.0365021111**	**-4.6354281848**	**-2.036910383**
**H**	**1.0237893491**	**-1.8659742287**	**-1.39522899**
**H**	**0.933563545**	**-0.378614238**	**-2.3504020417**
**H**	**2.5598340276**	**-0.1088530201**	**-0.4893640265**
**H**	**1.7552200062**	**-0.1789025492**	**1.889603359**
**H**	**1.5353411128**	**-1.7431202472**	**1.1053034603**
**H**	**-0.5340686885**	**-1.1017944144**	**2.3480863731**
**H**	**-2.1657923961**	**-1.3574265215**	**0.4917038293**
**H**	**-0.7982990364**	**-2.4367995116**	**0.2691501108**
**H**	**1.2503954279**	**1.8943702663**	**-1.2185861133**
**H**	**1.5973172365**	**1.9777040793**	**0.508948846**
**H**	**-0.8011019174**	**2.5439680832**	**0.057573605**
**H**	**-2.3350638179**	**0.7852174499**	**0.8486493701**
**H**	**-1.0808899478**	**1.2114223405**	**-2.0225896528**
**H**	**-0.2277482282**	**1.3862752123**	**2.212304516**
**H**	**-1.8327959006**	**0.9153755588**	**1.653415348**
**H**	**-0.7939394961**	**0.326301339**	**-6.1735172135**

(E_2) Sum of electronic and zero-point Energies = -3463.515119 Hartree

Scheme S2. Mulliken charge distribution for 15a and 15b at the RB3LYP/6-31-G(d,p)

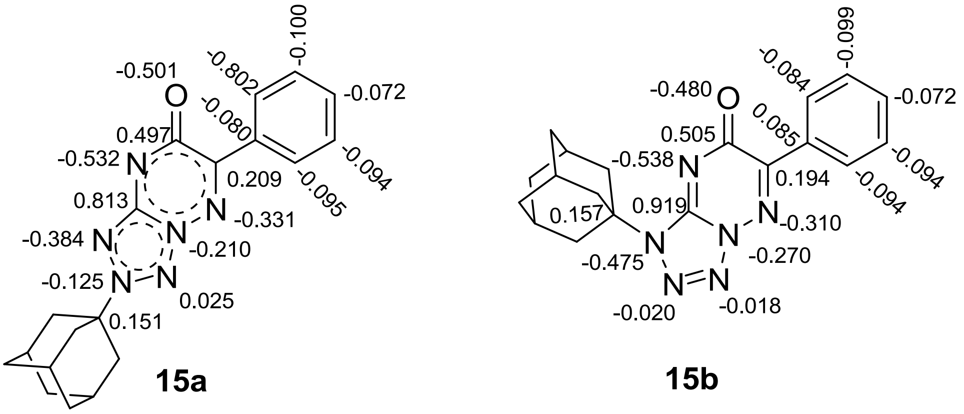

References

S1. Harris, R. K.; Becker, E. D.; Cabral de Menezes, S. M.; Goodfellow, R.; Granger, P. *Pure Appl. Chem*. **2001**, *73*, 1795-1818.

S2. Bax, A.; Vuister, G. W.; Grzesiek, S.; Delaglio, F.; Wang, A. C.; Tschudin, R.; Zhu, G. *Methods Enzymol.* **1994**, *239*, 79－105.

S3. Deev, S. L.; Shenkarev, Z. O.; Shestakova, T. S.; Chupakhin, O. N.; Rusinov, V. L.; Arseniev, A. S. *J. Org. Chem*. **2010**, *75*, 8487-8497.

S4. Shestakova, T. S.; Shenkarev, Z. O.; Deev, S. L.; Chupakhin, O. N.; Khalymbadzha, I. A.; Rusinov, V. L.; Arseniev, A. S. *J. Org. Chem*. **2013**, *78*, 6975–6982

S5. Kupče, Ē; Freeman, R. *J. Magn. Reson., Ser. A.* **1996**, *118*, 299－303.

S6. Sheldrick, G. M. *SHELXS-97, a Program for the Automatic Solution of Crystal Structures*; University of Gӧttingen, Gӧttingen, Germany, 1997.

S7. Sheldrick, G. M. *SHELXL-97, a Program for Crystal Structure Refinement*; University of Gӧttingen, Gӧttingen, Germany, 1997.

S8. Khalymbadzha, I. A.; Shestakova, T. S.; Deev, S. L.; Rusinov, V. L.; Chupakhin, O. N.; Shenkarev, Z. O.; Arseniev, A. S. *Russ. Chem. Bull.* **2013**, *62*, 521-528.

S9. Deev, S. L.; Shenkarev, Z. O.; Shestakova, T. S.; Chupakhin, O. N.; Rusinov, V. L.; Arseniev, A. S. *J. Org. Chem*. **2010**, *75*, 8487-8497.

S10. Chupakhin, O. N.; Ulomsky, E. N.; Deev, S. L.; Rusinov, V. L. *Synth. Commun*. **2001**, *31*, 2351-2355.

S11. Maquestiau, A.; Van Haverbeke, Y.; Flammang, R. *Tetrahedron Lett.*, **1976**, *41*, 3747-3750.

S12. Gaussian 09, Revision D.01, M. J. Frisch, G. W. Trucks, H. B. Schlegel, G. E. Scuseria, . A. Robb, J. R. Cheeseman, G. Scalmani, V. Barone, B. Mennucci, G. A. Petersson, H. Nakatsuji, M. Caricato, X. Li, H. P. Hratchian, A. F. Izmaylov, J. Bloino, G. Zheng, J. L. Sonnenberg, M. Hada, M. Ehara, K. Toyota, R. Fukuda, J. Hasegawa, M. Ishida, T. Nakajima, Y. Honda, O. Kitao, H. Nakai, T. Vreven, J. A. Montgomery, Jr., J. E. Peralta, F. Ogliaro, M. Bearpark, J. J. Heyd, E. Brothers, K. N. Kudin, V. N. Staroverov, T. Keith, R. Kobayashi, J. Normand, K. Raghavachari, A. Rendell, J. C. Burant, S. S. Iyengar, J. Tomasi, M. Cossi, N. Rega, J. M. Millam, M. Klene, J. E. Knox, J. B. Cross, V. Bakken, C. Adamo, J. Jaramillo, R. Gomperts, R. E. Stratmann, O. Yazyev, A. J. Austin, R. Cammi, C. Pomelli, J. W. Ochterski, R. L. Martin, K. Morokuma, V. G. Zakrzewski, G. A. Voth, P. Salvador, J. J. Dannenberg, S. Dapprich, A. D. Daniels, O. Farkas, J. B. Foresman, J. V. Ortiz, J. Cioslowski, and D. J. Fox, Gaussian, Inc., Wallingford CT, **2013**.

NMR spectra

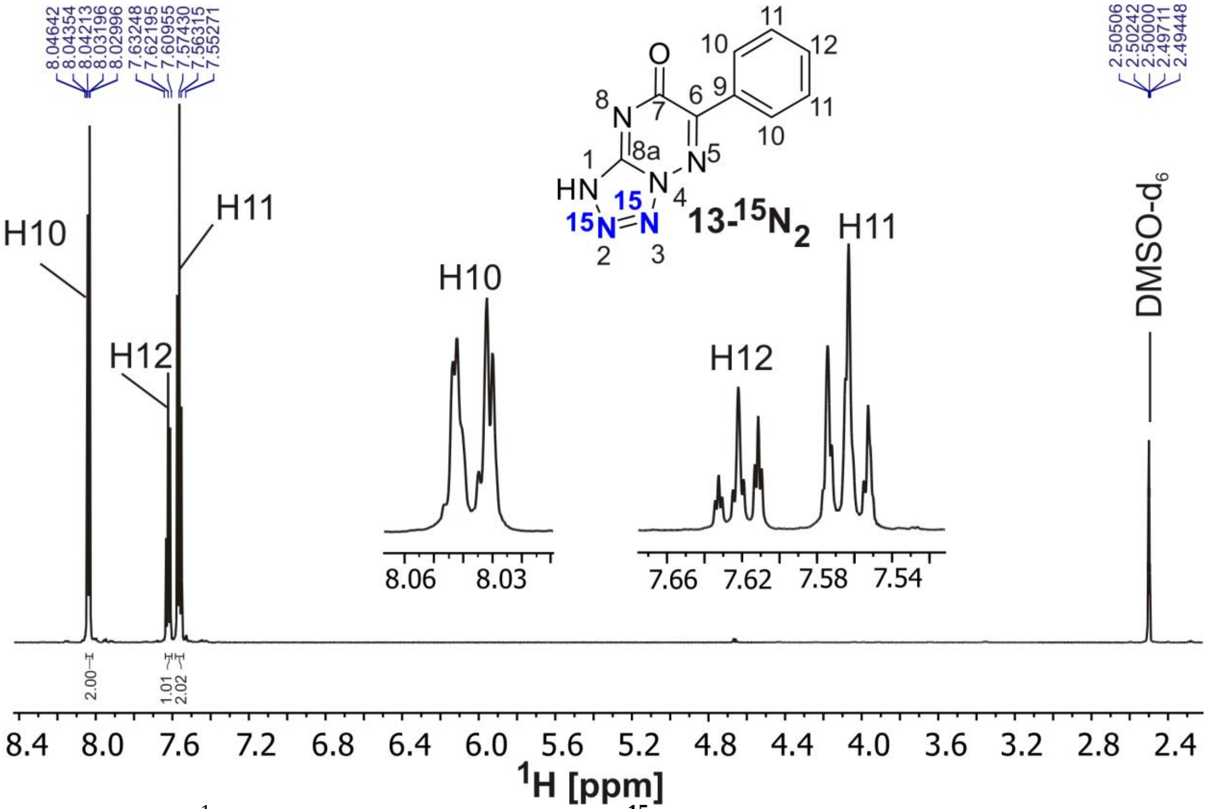

Figure S1: 1D ^{1}H NMR spectrum of 70 mM **13-^{15}N$_2$** measured in DMSO-d_6 (700 MHz, 45 °C). The resonance assignment is shown.

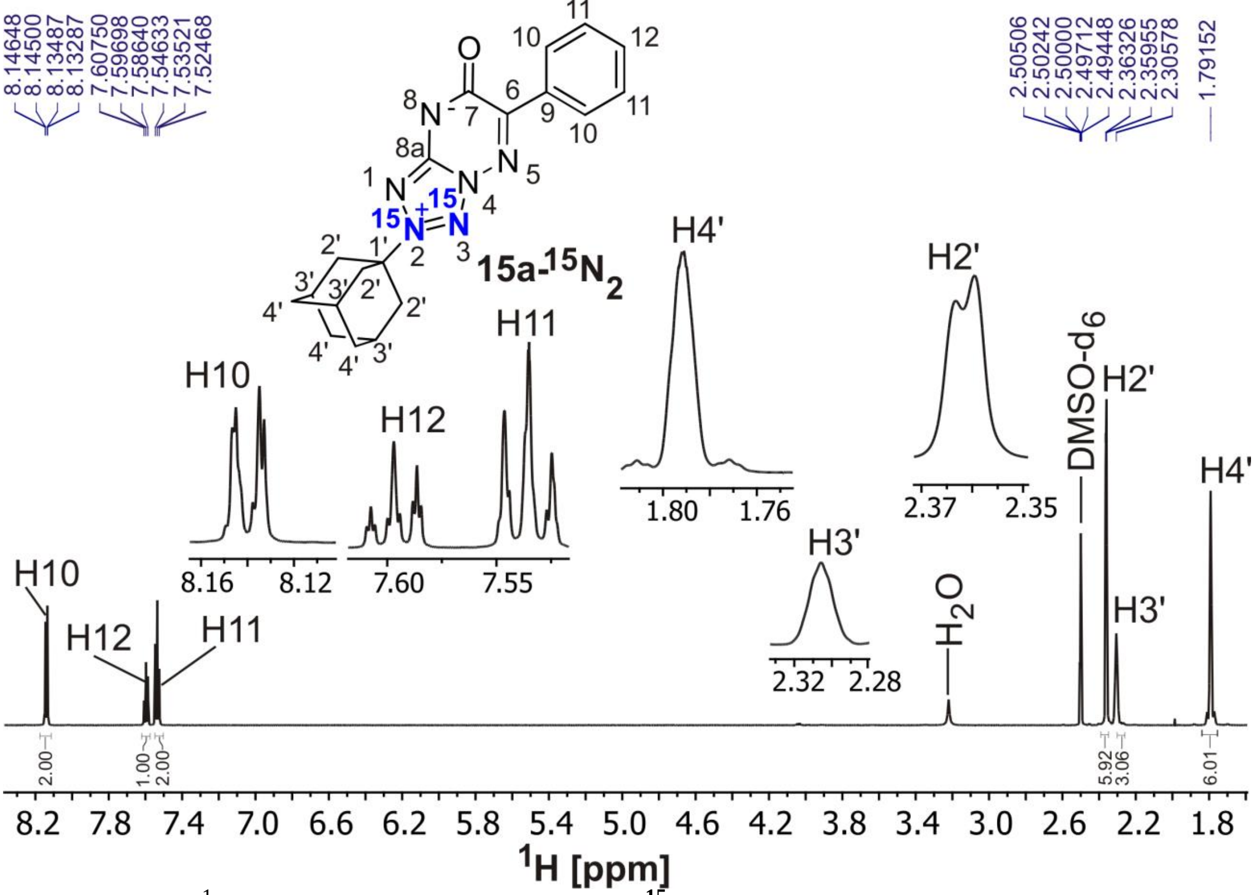

Figure S2: 1D ^{1}H NMR spectrum of 35 mM **15a-^{15}N$_2$** measured in DMSO-d_6 (700 MHz, 45 °C). The resonance assignment is shown.

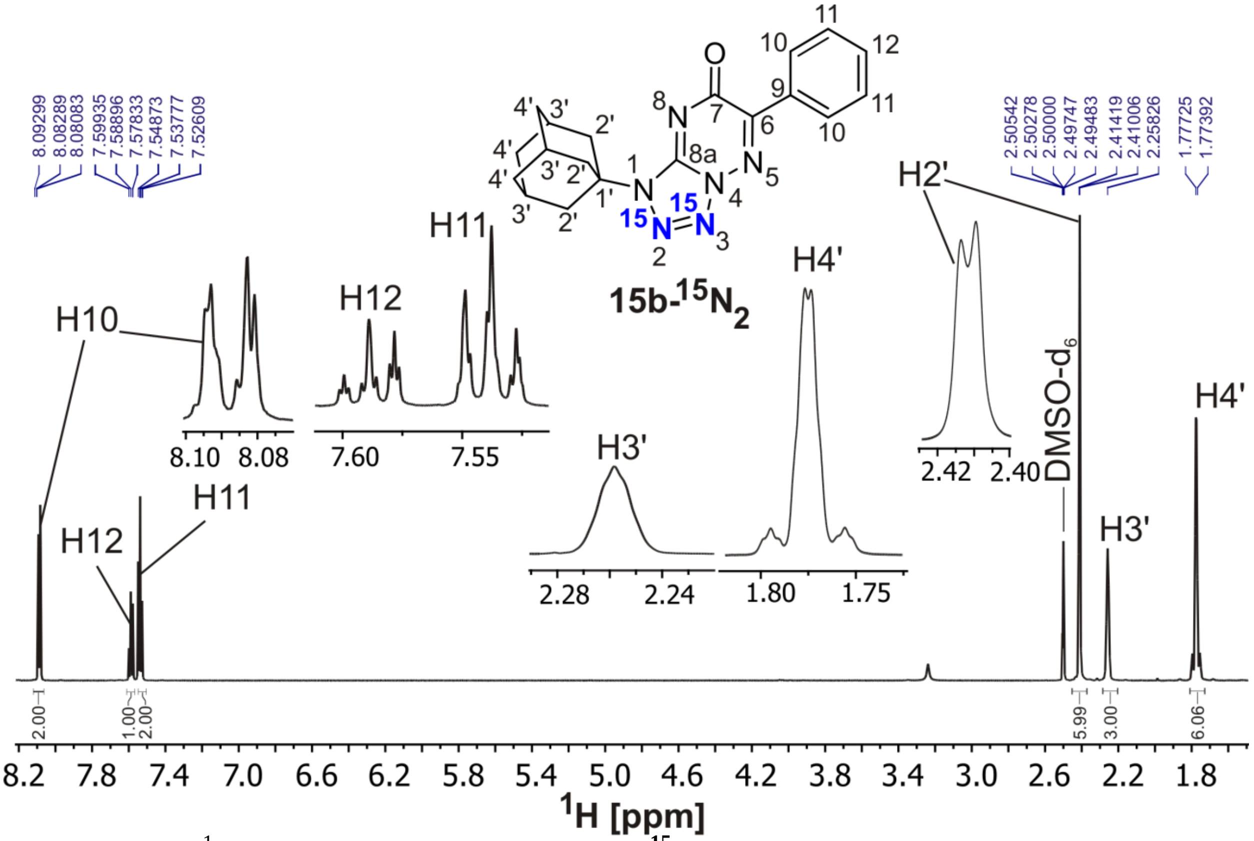

Figure S3: 1D ^{1}H NMR spectrum of 30 mM **15b-^{15}N$_2$** measured in DMSO-d_6 (700 MHz, 45 °C). The resonance assignment is shown.

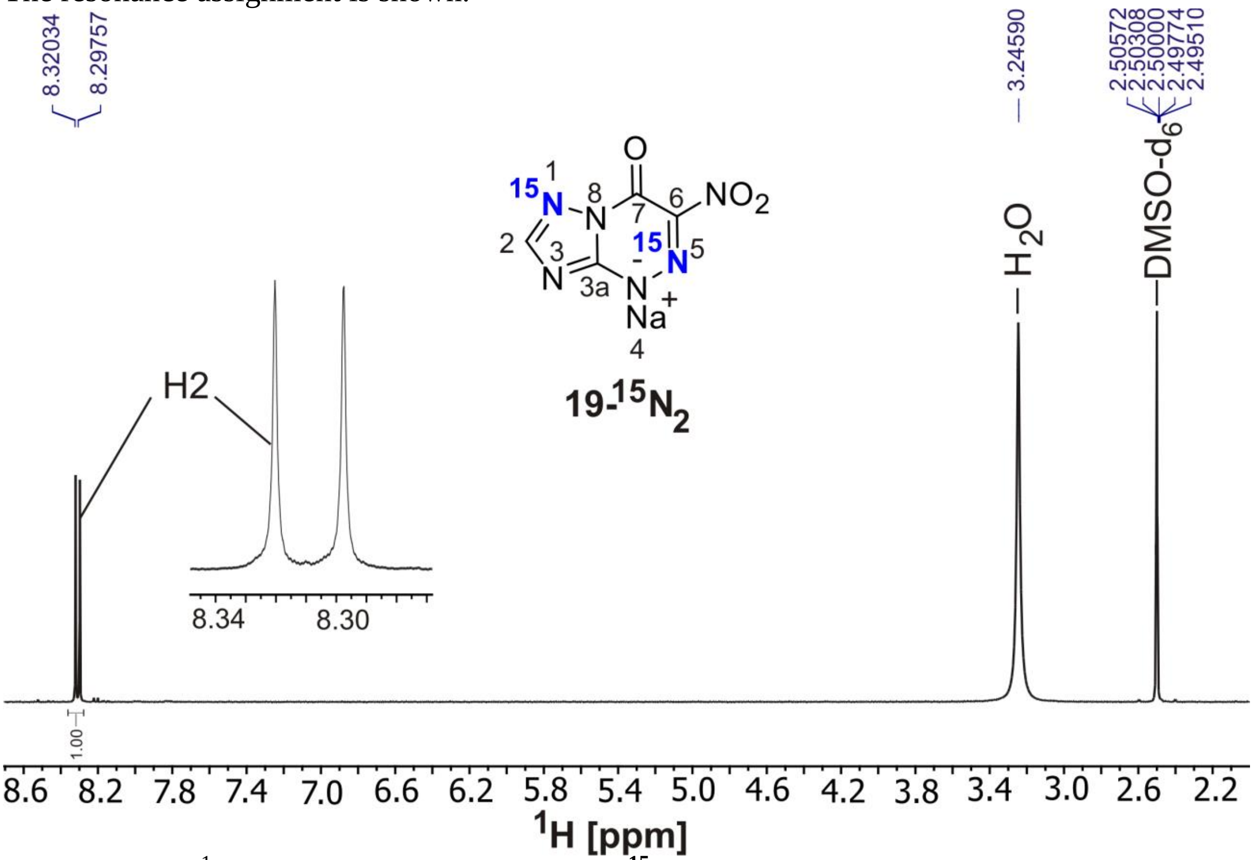

Figure S4: 1D ^{1}H NMR spectrum of 36 mM **19-^{15}N$_2$** measured in DMSO-d_6 (700 MHz, 45 °C). The resonance assignment is shown.

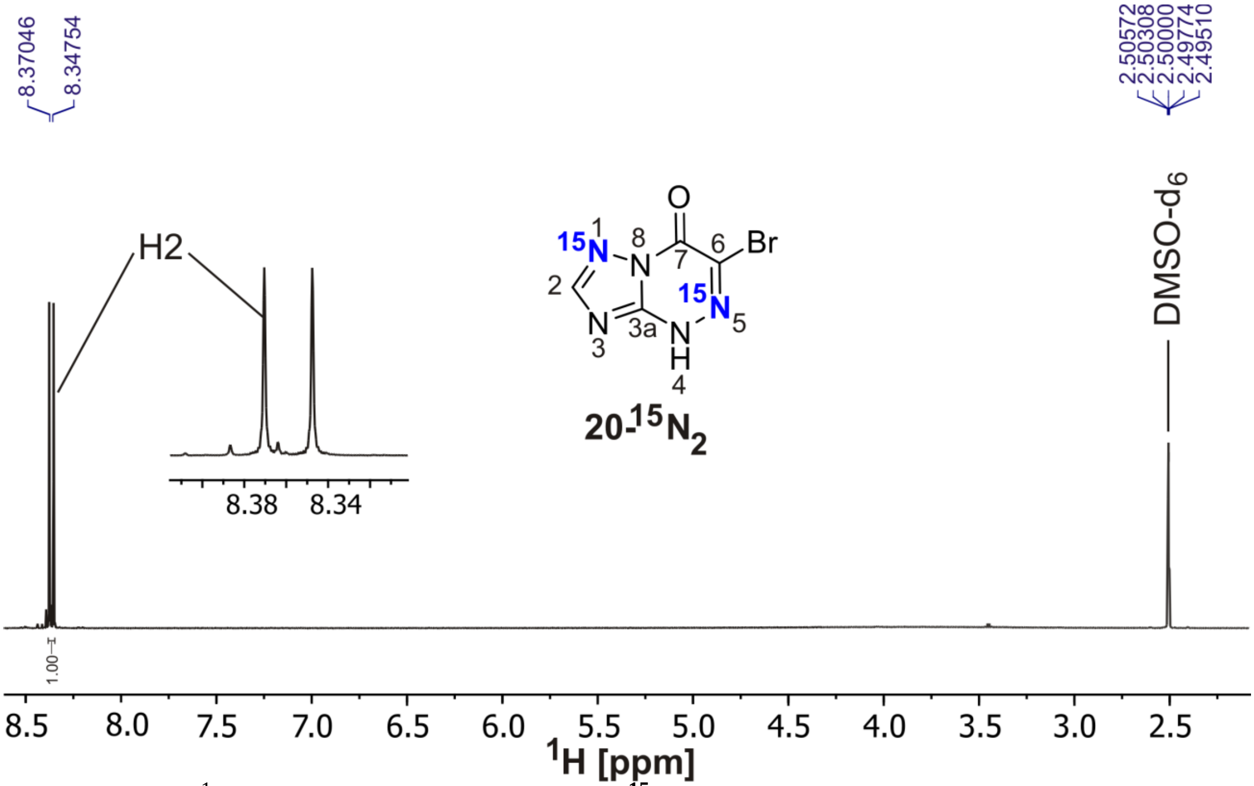

Figure S5: 1D ^{1}H NMR spectrum of 46 mM **20-^{15}N$_2$** measured in DMSO-d_6 (700 MHz, 45 °C). The resonance assignment is shown.

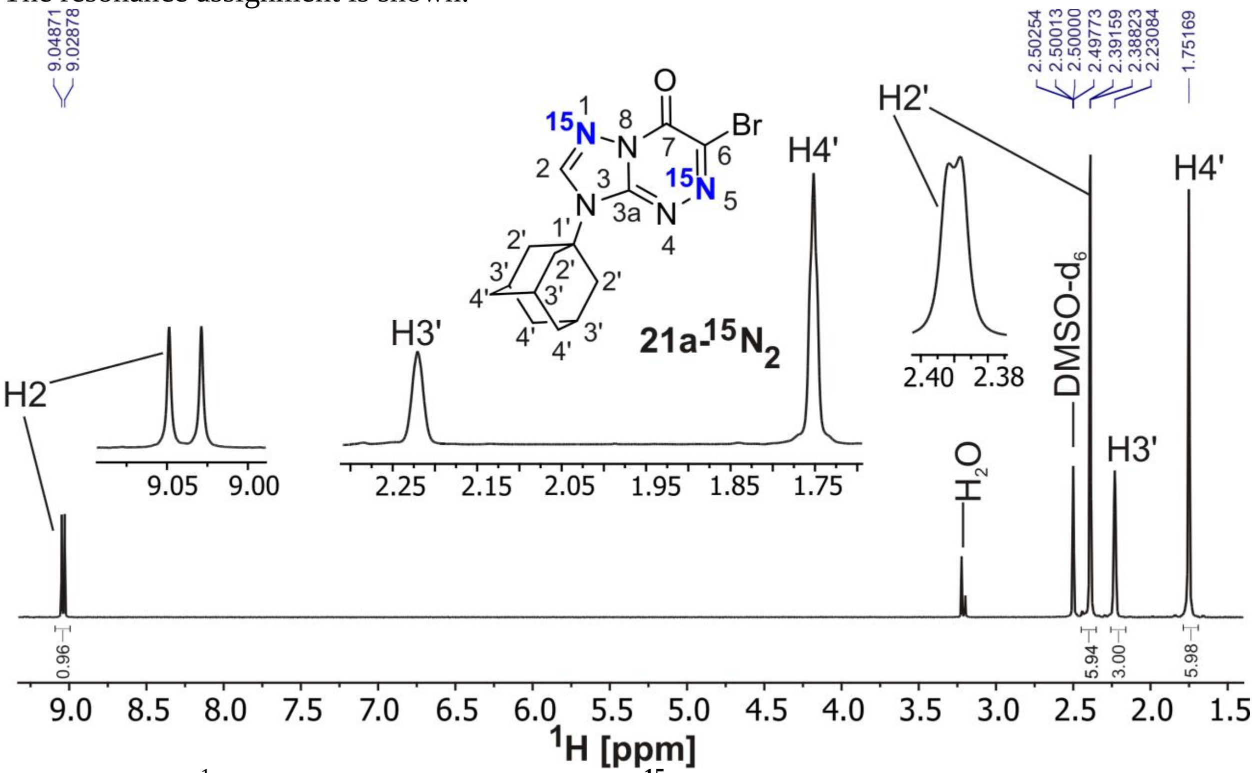

Figure S6: 1D ^{1}H NMR spectrum of 36 mM **21a-^{15}N$_2$** measured in DMSO-d_6 (700 MHz, 45 °C). The resonance assignment is shown.

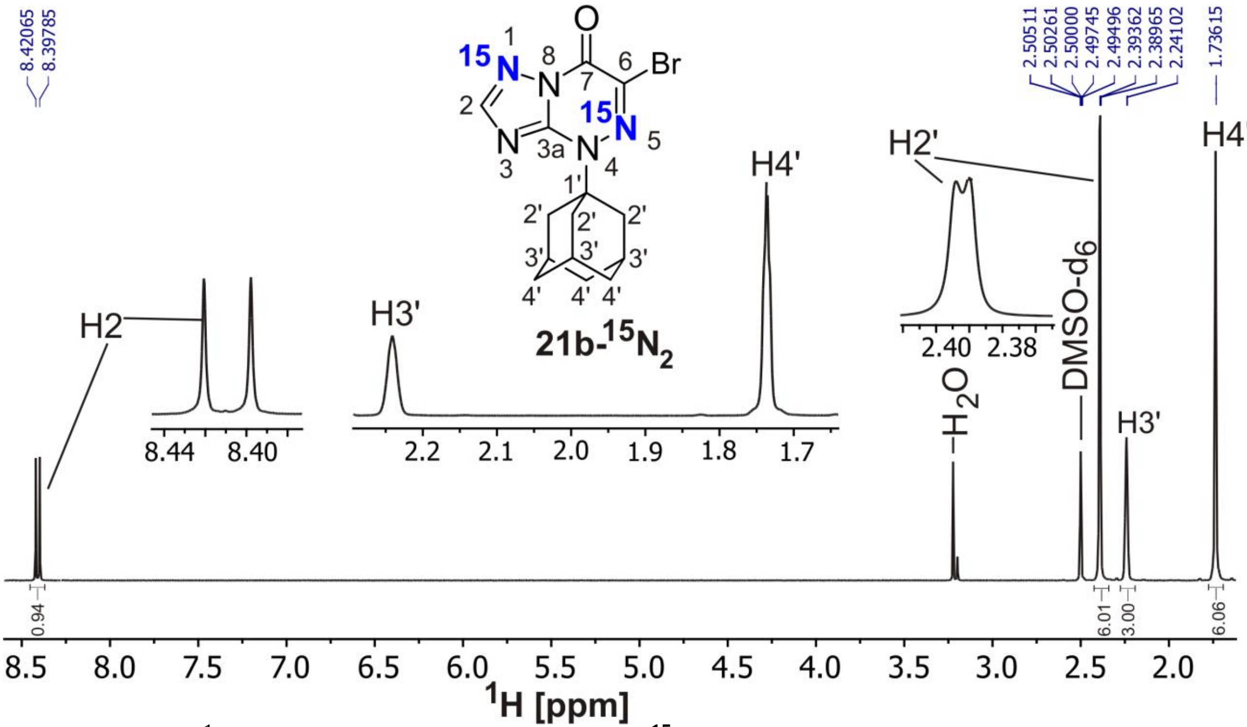

Figure S7: 1D ^{1}H NMR spectrum of 40 mM **21b-^{15}N$_2$** measured in DMSO-d_6 (700 MHz, 45 °C). The resonance assignment is shown.

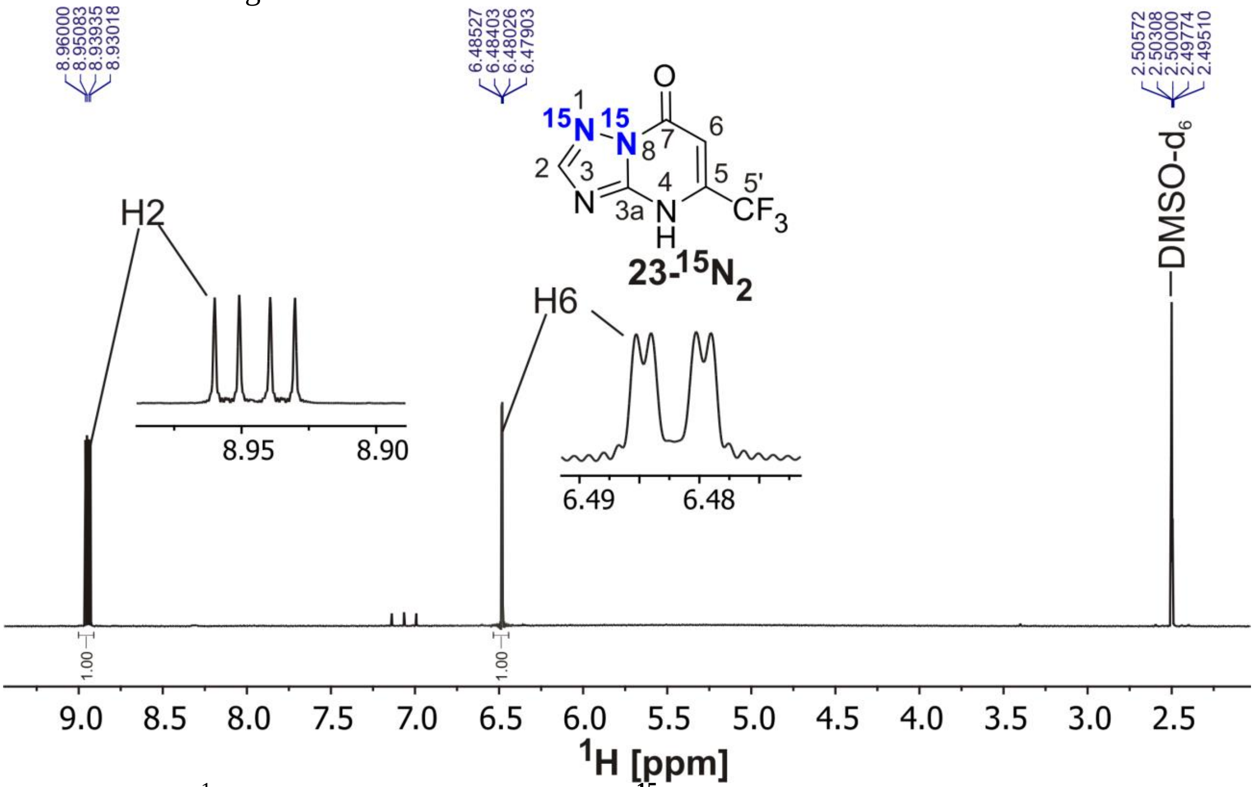

Figure S8: 1D ^{1}H NMR spectrum of 50 mM **23-^{15}N$_2$** measured in DMSO-d_6 (700 MHz, 45 °C). The resonance assignment is shown.

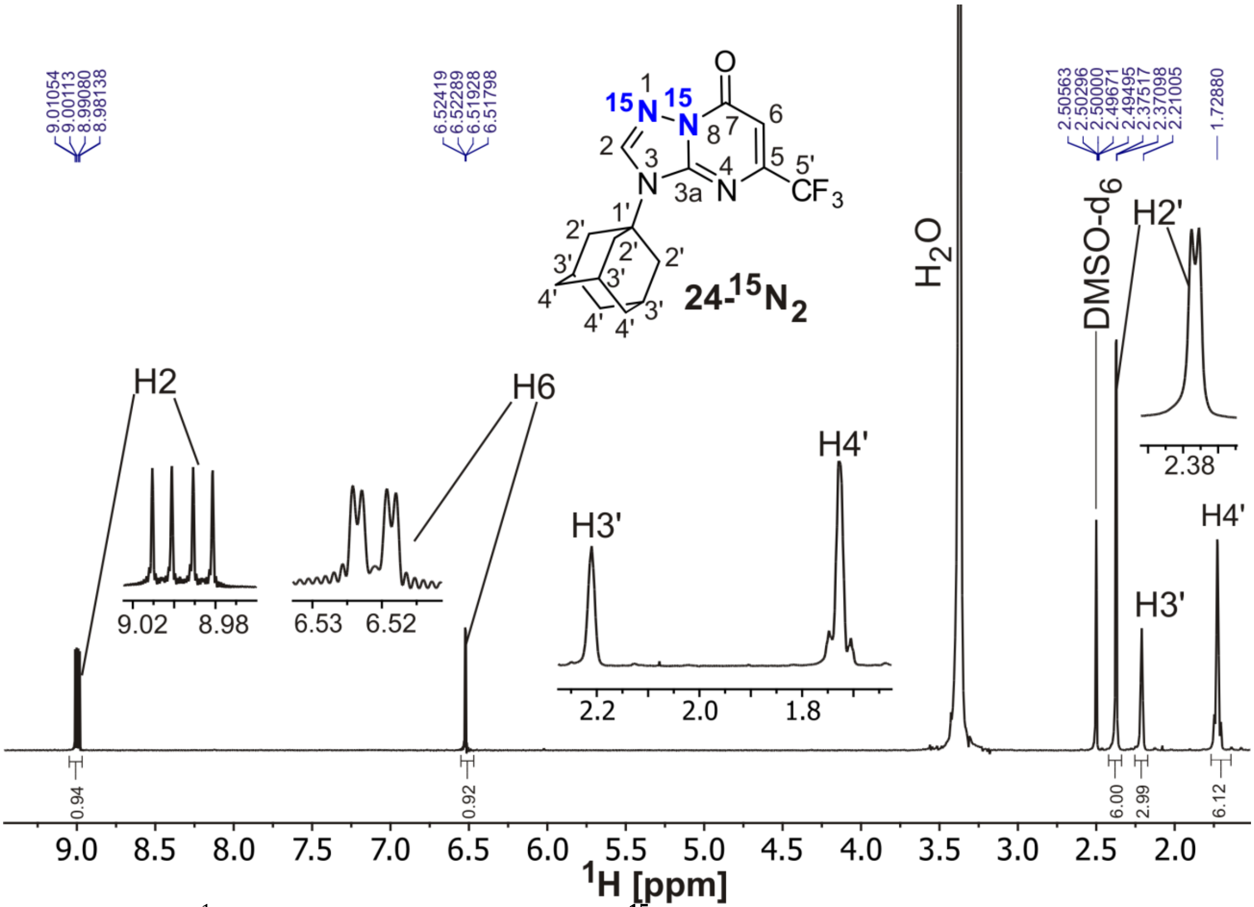

Figure S9: 1D ^{1}H NMR spectrum of 42 mM **24-^{15}N$_2$** measured in DMSO-d_6 (700 MHz, 45 °C). The resonance assignment is shown.

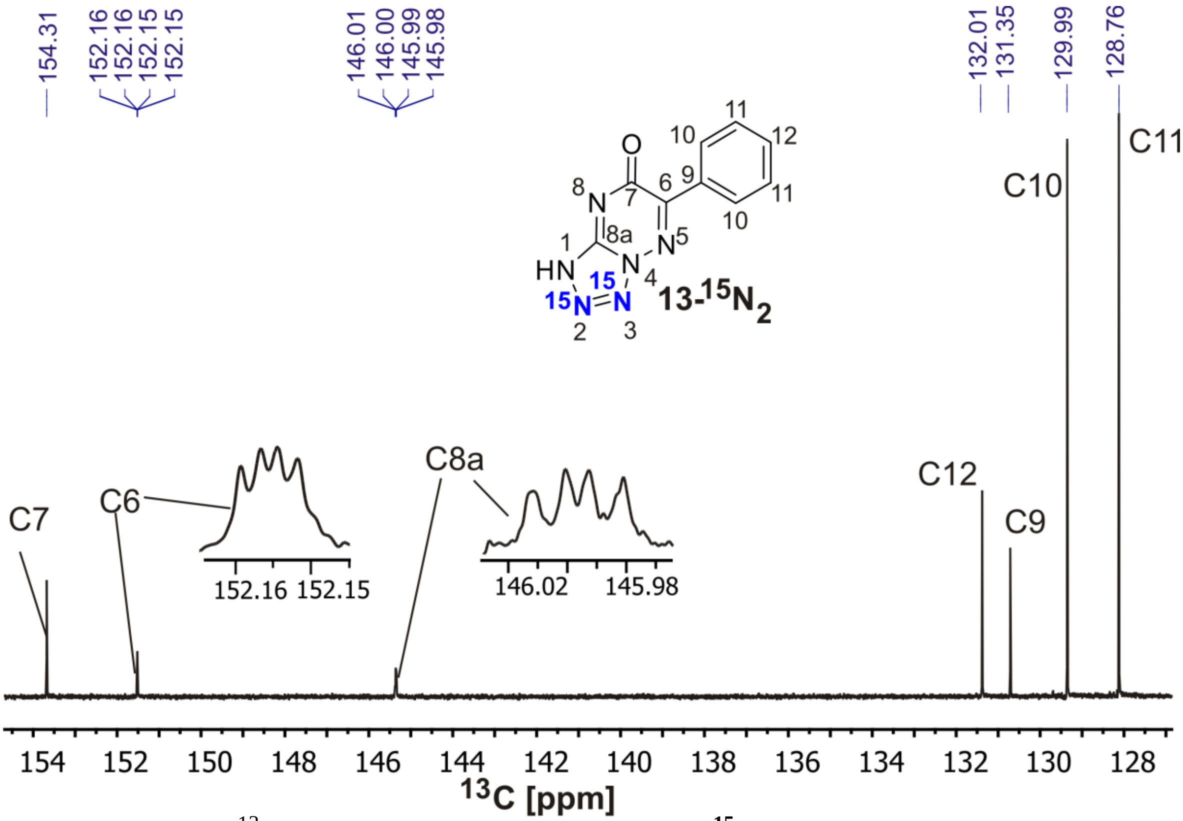

Figure S10: 1D ^{13}C NMR spectrum of 70 mM **13-^{15}N$_2$** measured in DMSO-d_6 (700 MHz, 45 °C). The resonance assignment is shown.

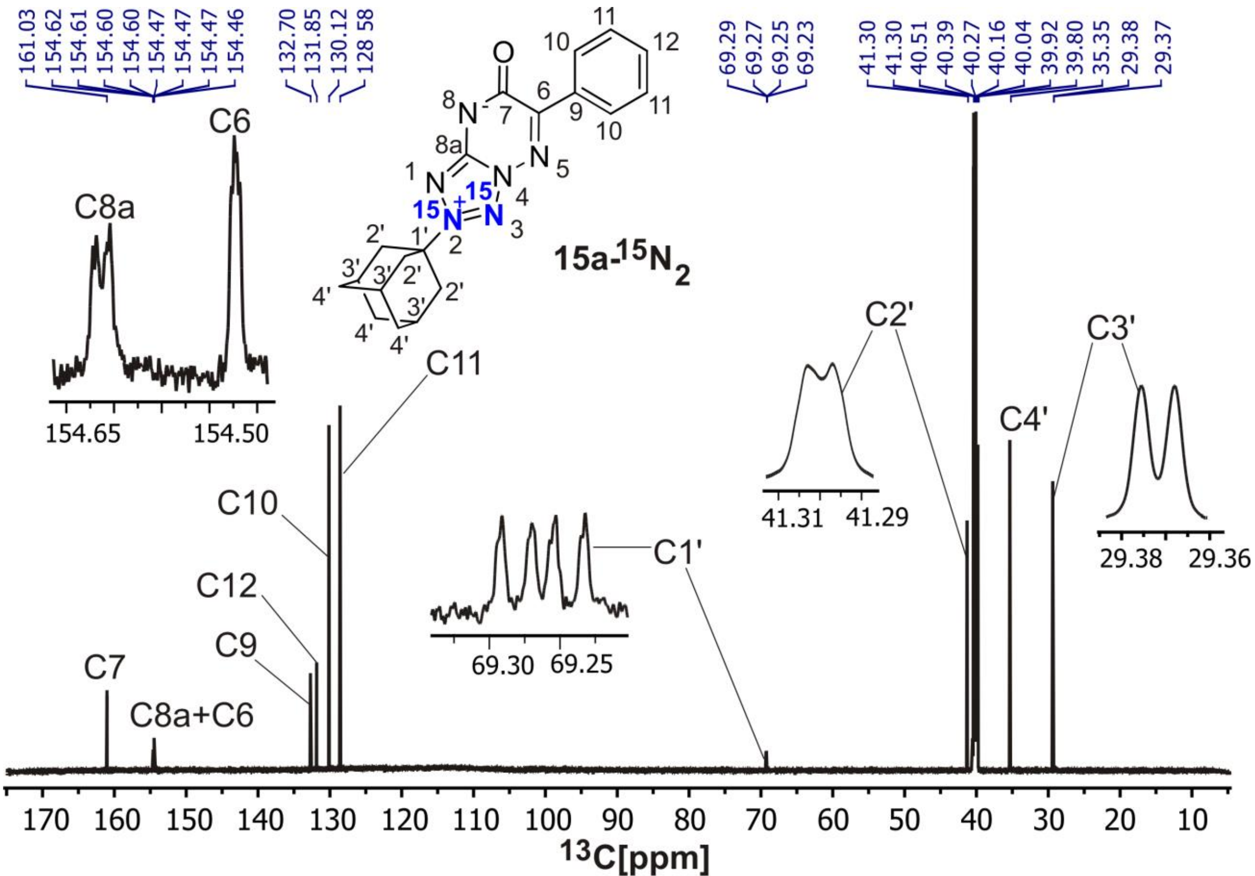

Figure S11: 1D ^{13}C NMR spectrum of 35 mM **15a-$^{15}N_2$** measured in DMSO-d_6 (700 MHz, 45 °C). The resonance assignment is shown.

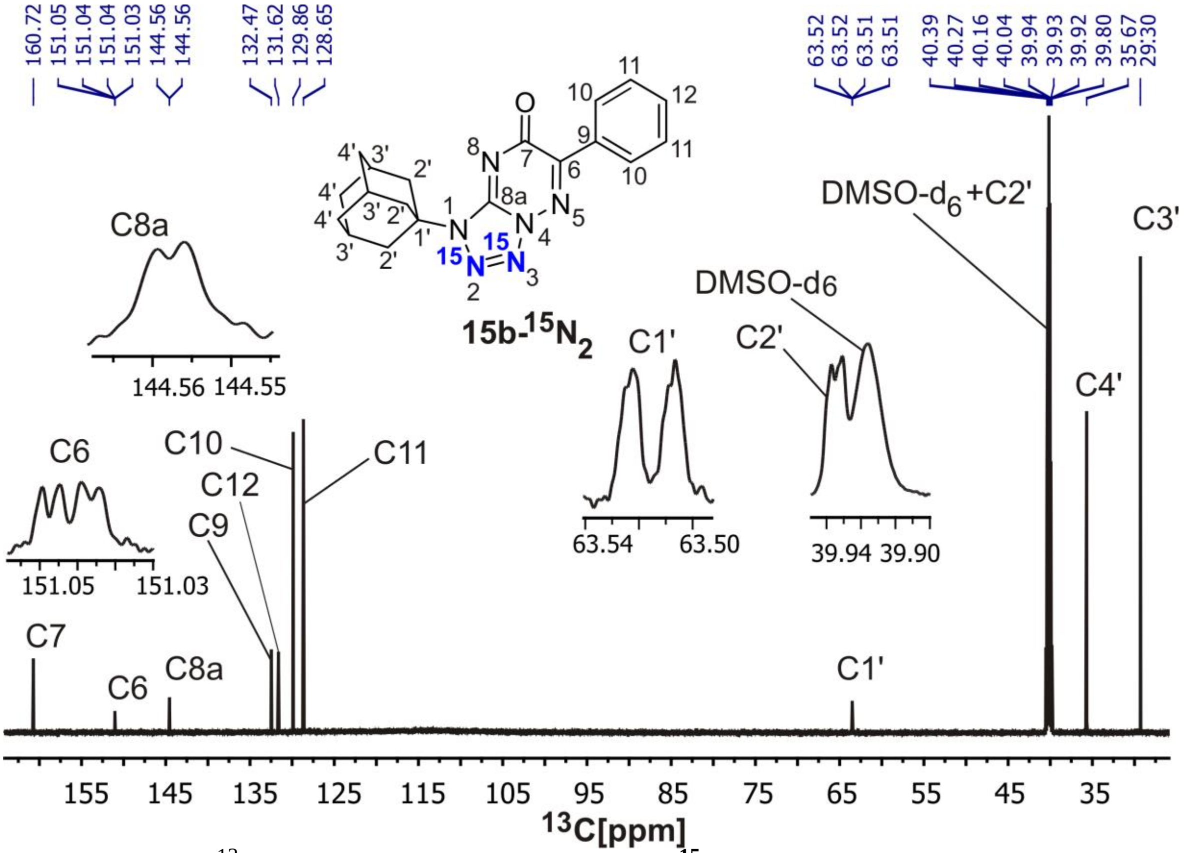

Figure S12: 1D ^{13}C NMR spectrum of 30 mM **15b-$^{15}N_2$** measured in DMSO-d_6 (700 MHz, 45 °C). The resonance assignment is shown.

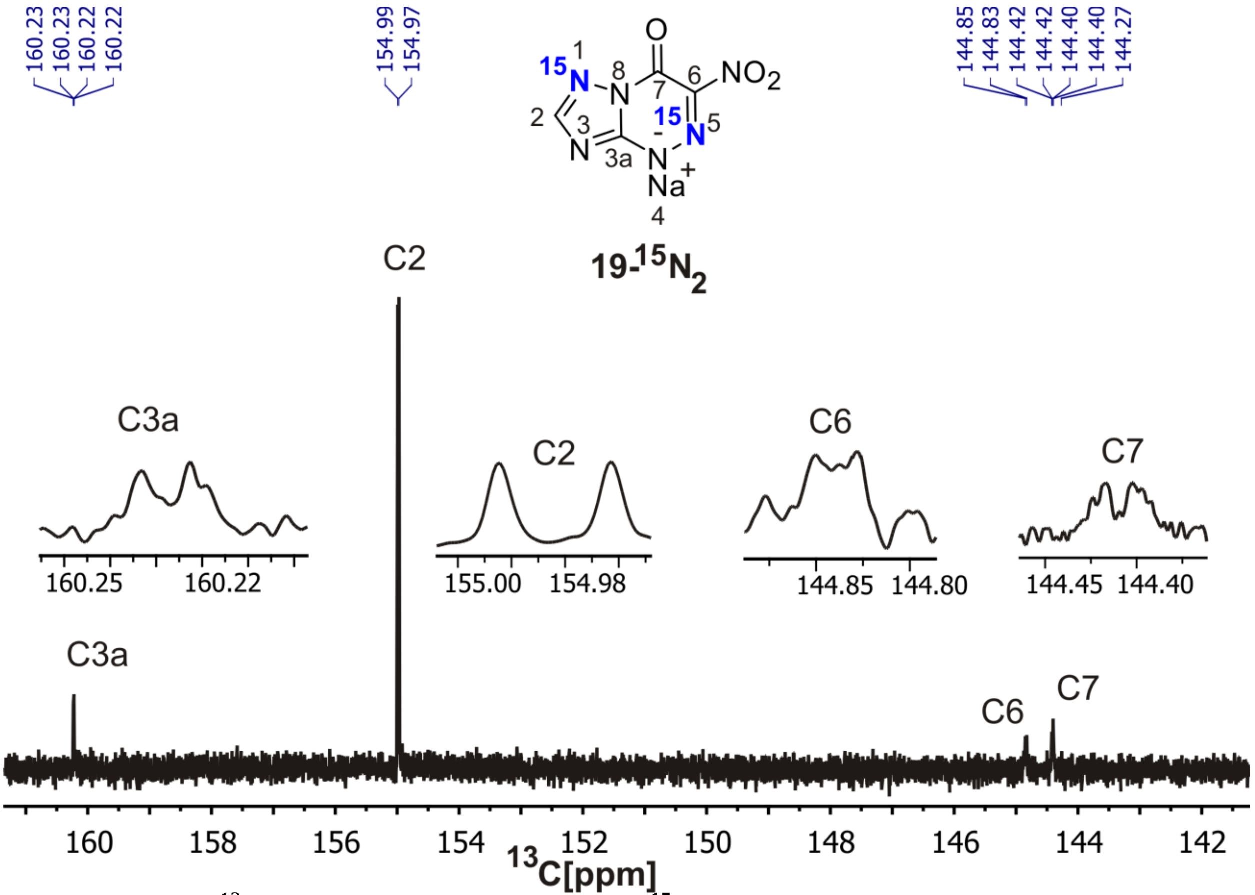

Figure S13: 1D ^{13}C NMR spectrum of 36mM **19-^{15}N$_2$** measured in DMSO-d_6 (700 MHz, 45 °C). The resonance assignment is shown.

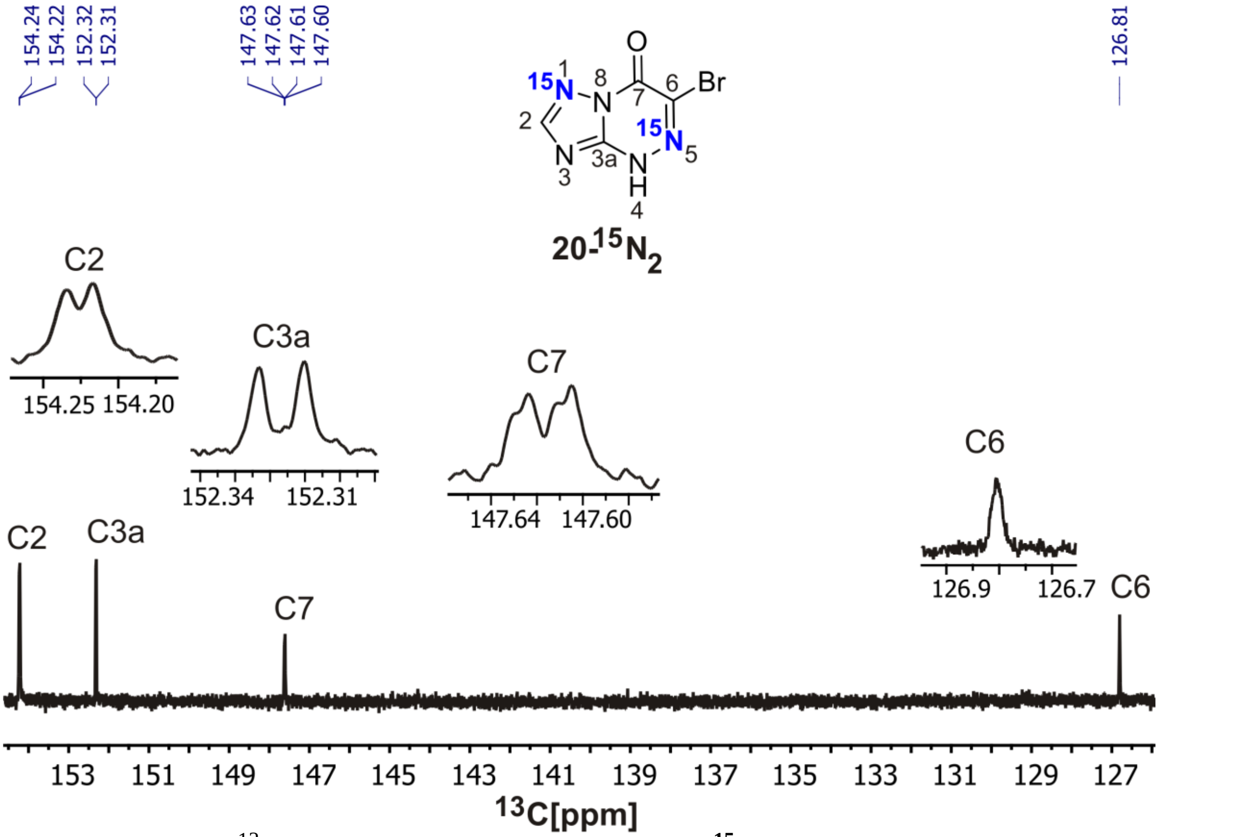

Figure S14: 1D ^{13}C NMR spectrum of 46 mM **20-^{15}N$_2$** measured in DMSO-d_6 (700 MHz, 45 °C). The resonance assignment is shown.

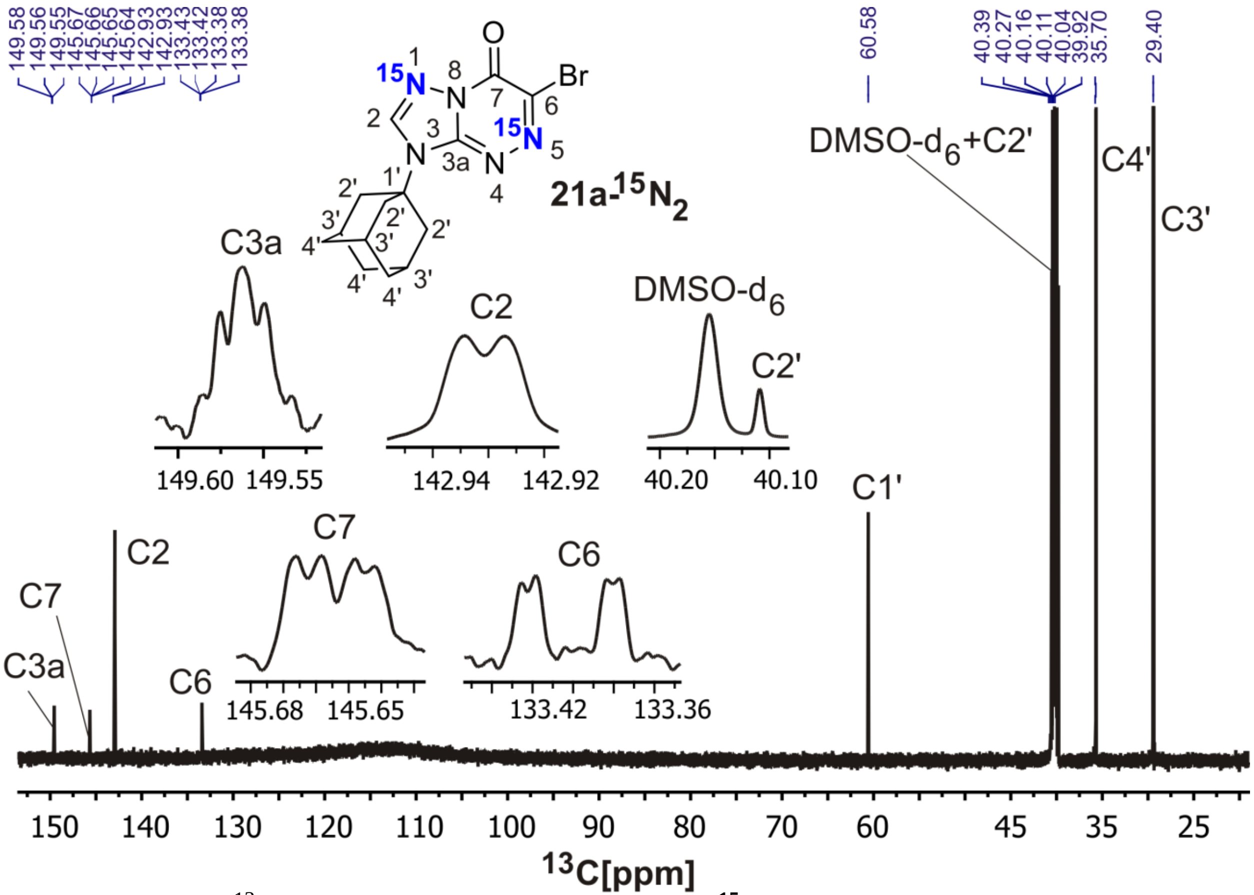

Figure S15: 1D ^{13}C NMR spectrum of 36 mM **21a-^{15}N$_2$** measured in DMSO-d_6 (700 MHz, 45 °C). The resonance assignment is shown.

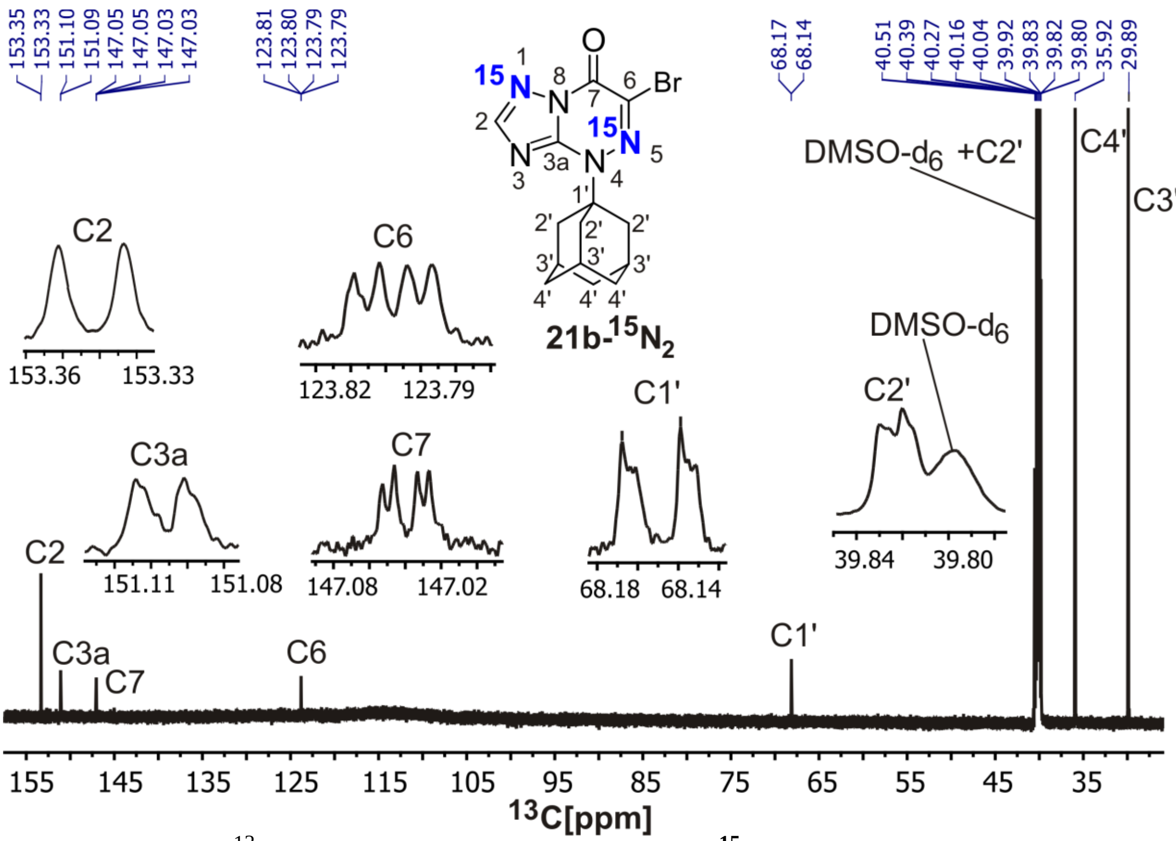

Figure S16: 1D ^{13}C NMR spectrum of 40 mM **21b-^{15}N$_2$** measured in DMSO-d_6 (700 MHz, 45 °C). The resonance assignment is shown.

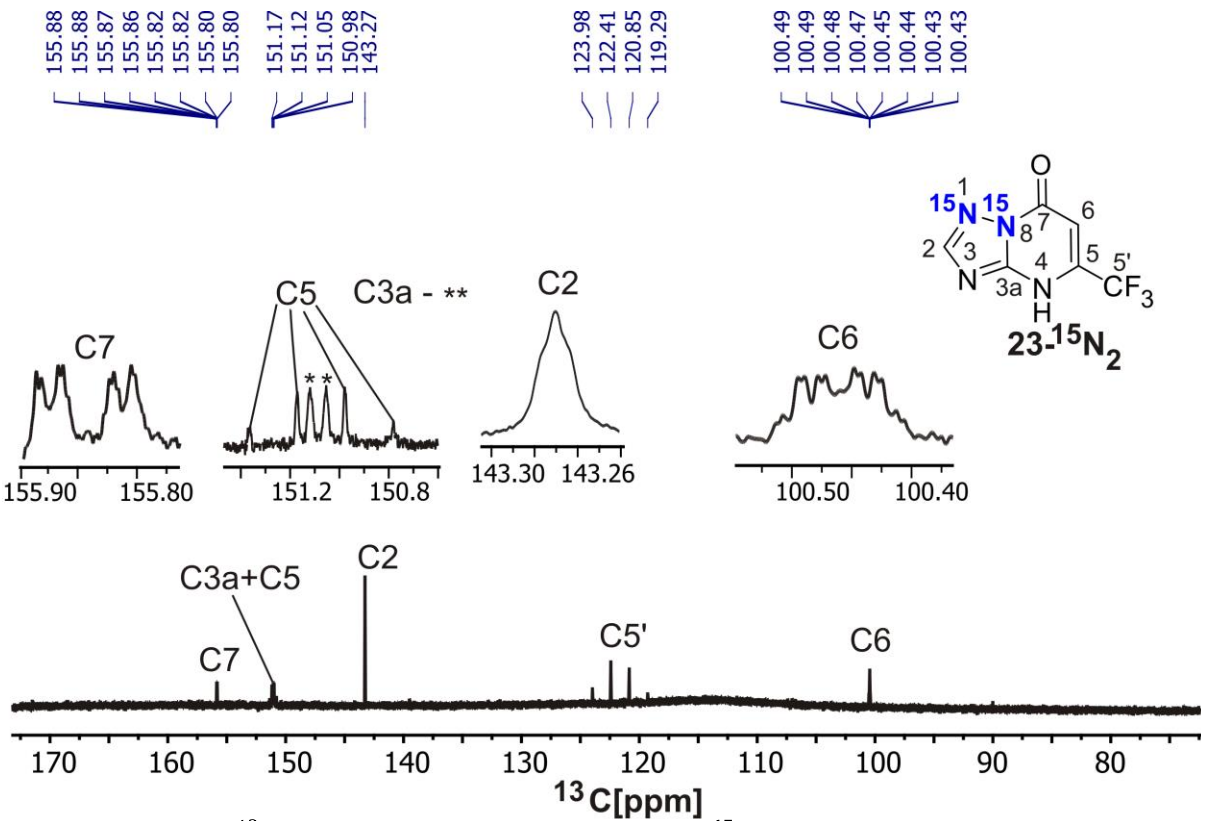

Figure S17: 1D ^{13}C NMR spectrum of 50 mM **23-^{15}N$_2$** measured in DMSO-d_6 (700 MHz, 45 °C). The resonance assignment is shown.

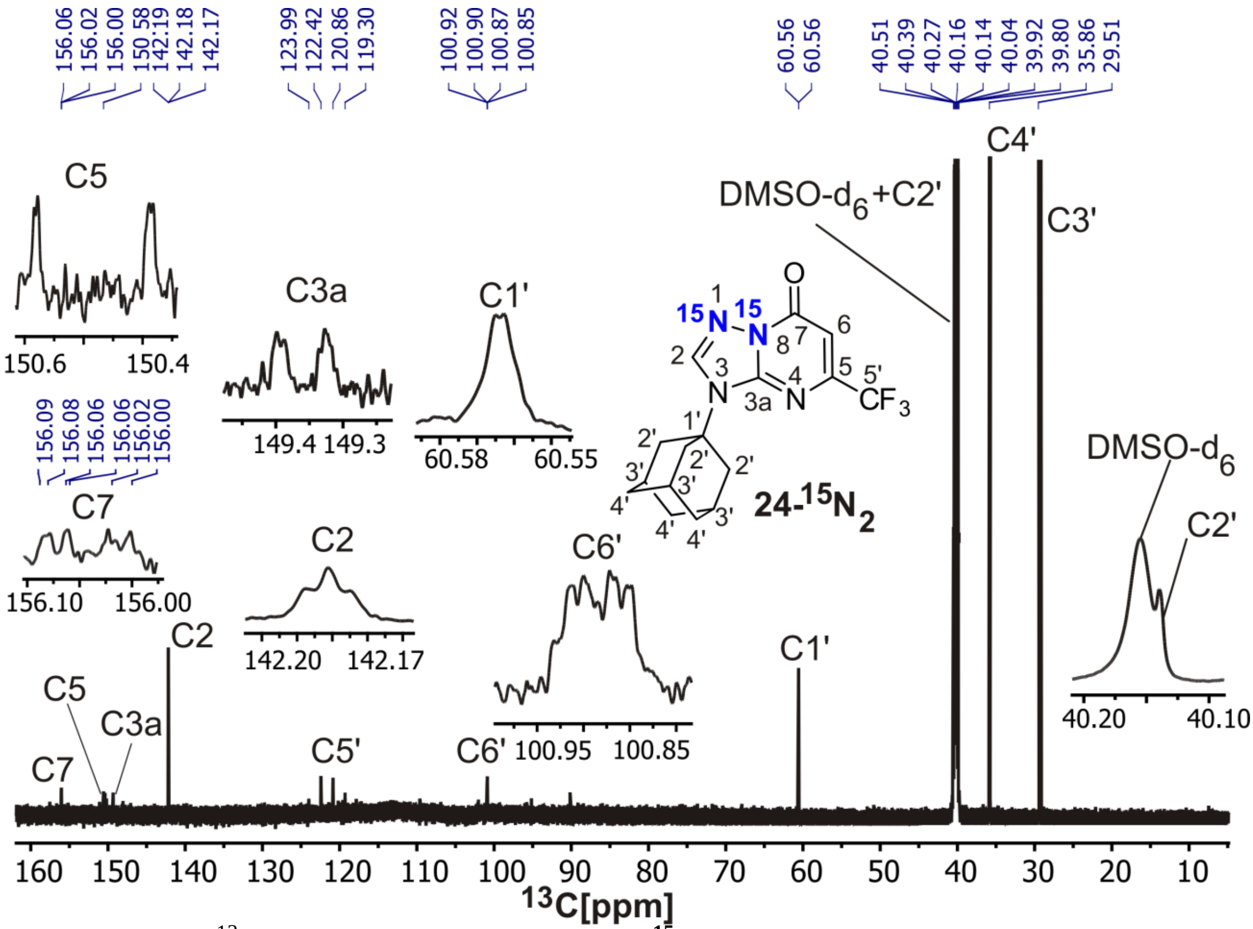

Figure S18: 1D ^{13}C NMR spectrum of 42 mM **24-^{15}N$_2$** measured in DMSO-d_6 (700 MHz, 45 °C). The resonance assignment is shown.

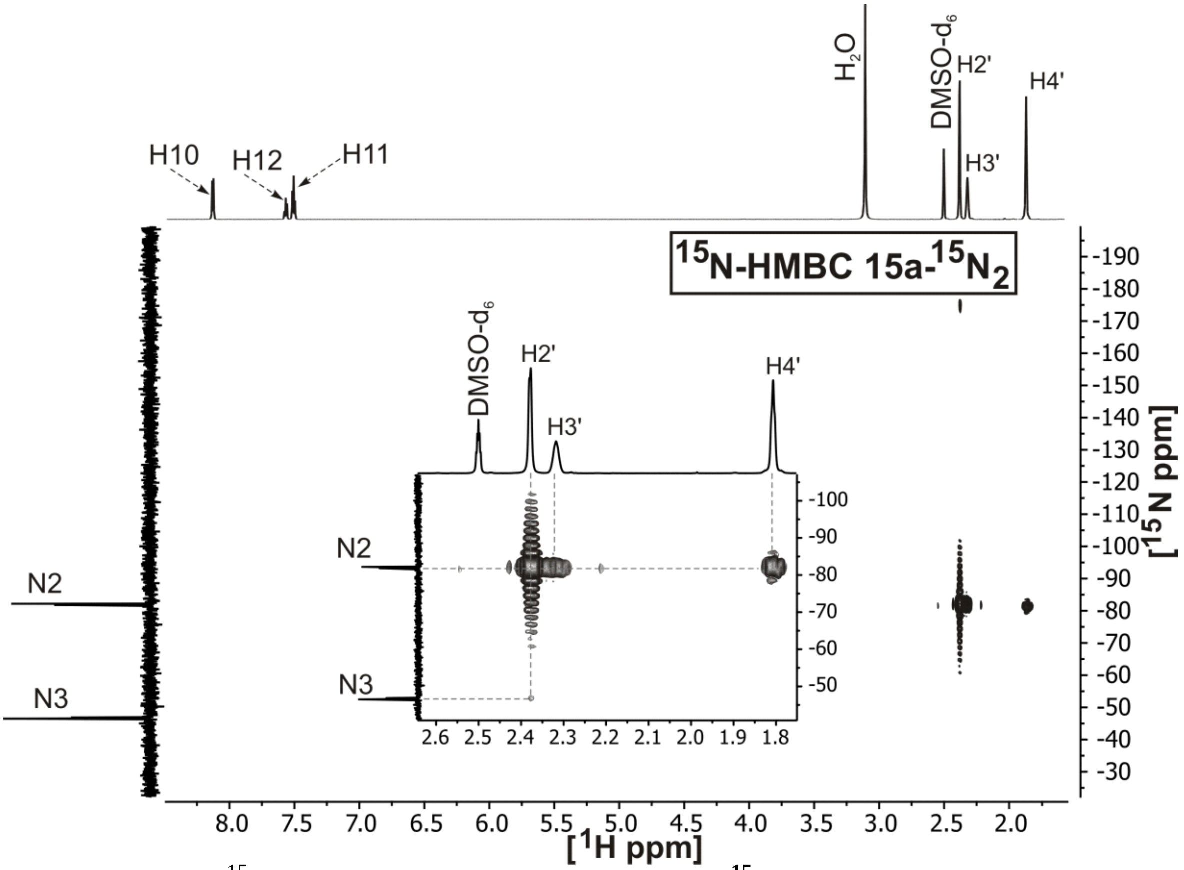

Figure S19: 2D ^{15}N HMBC spectrum of 51 mM **15a-^{15}N$_2$** measured in DMSO-d_6 (400 MHz, 45 °C). Inset shows the enlarged part of the spectrum with additional intensity scaling.

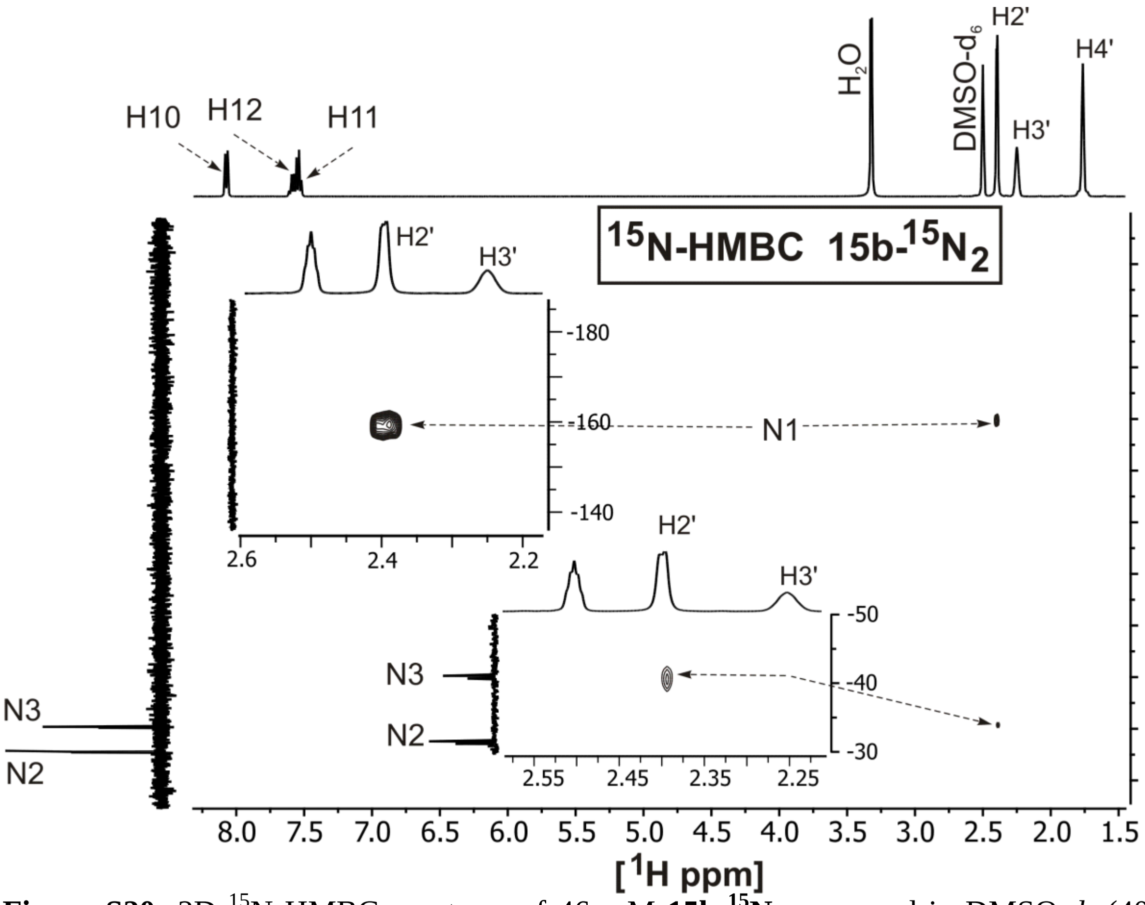

Figure S20: 2D ^{15}N HMBC spectrum of 46 mM **15b-^{15}N$_2$** measured in DMSO-d_6 (400 MHz, 45 °C). Inset shows the enlarged parts of the spectrum with additional intensity scaling.

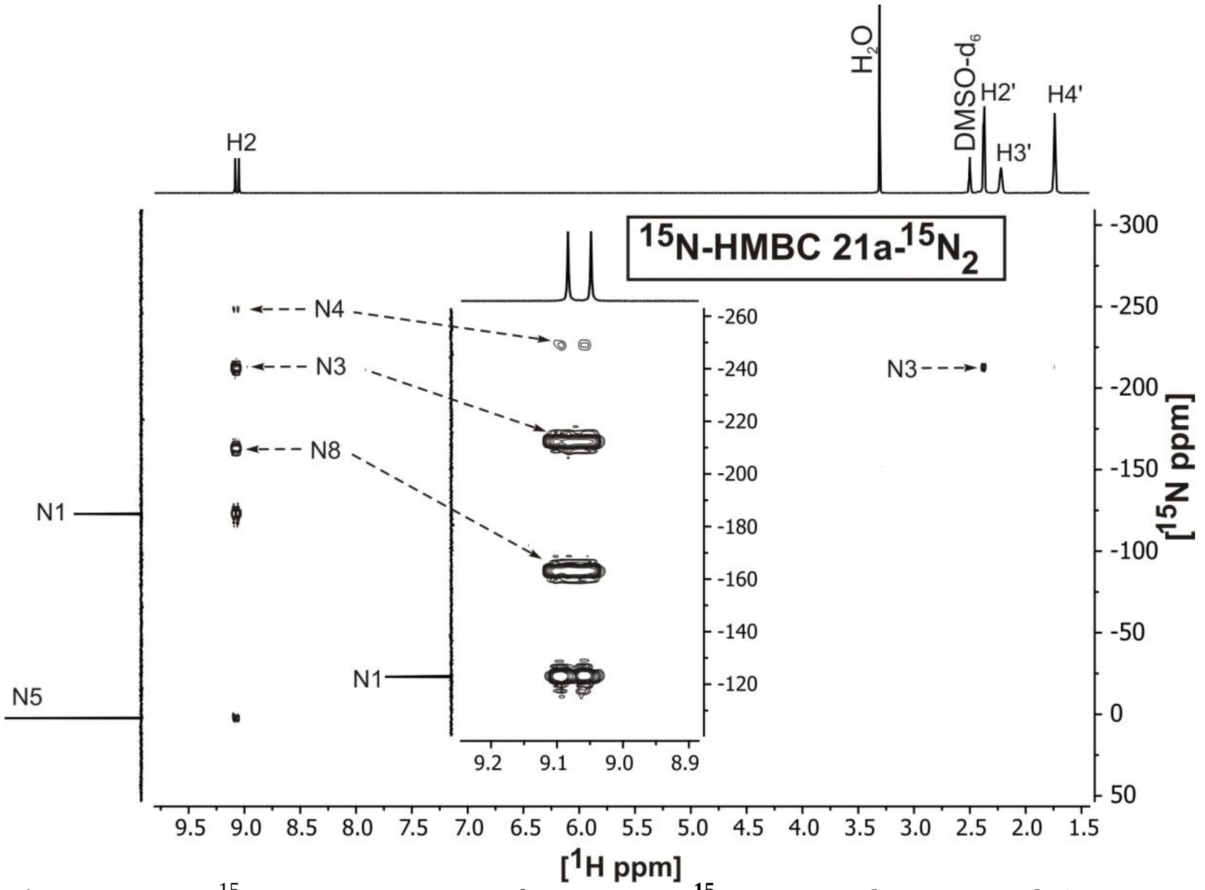

Figure S21: 2D ^{15}N HMBC spectrum of 40 mM **21a-^{15}N$_2$** measured in DMSO-d_6 (400 MHz, 45 °C). Inset shows the enlarged part of the spectrum with additional intensity scaling.

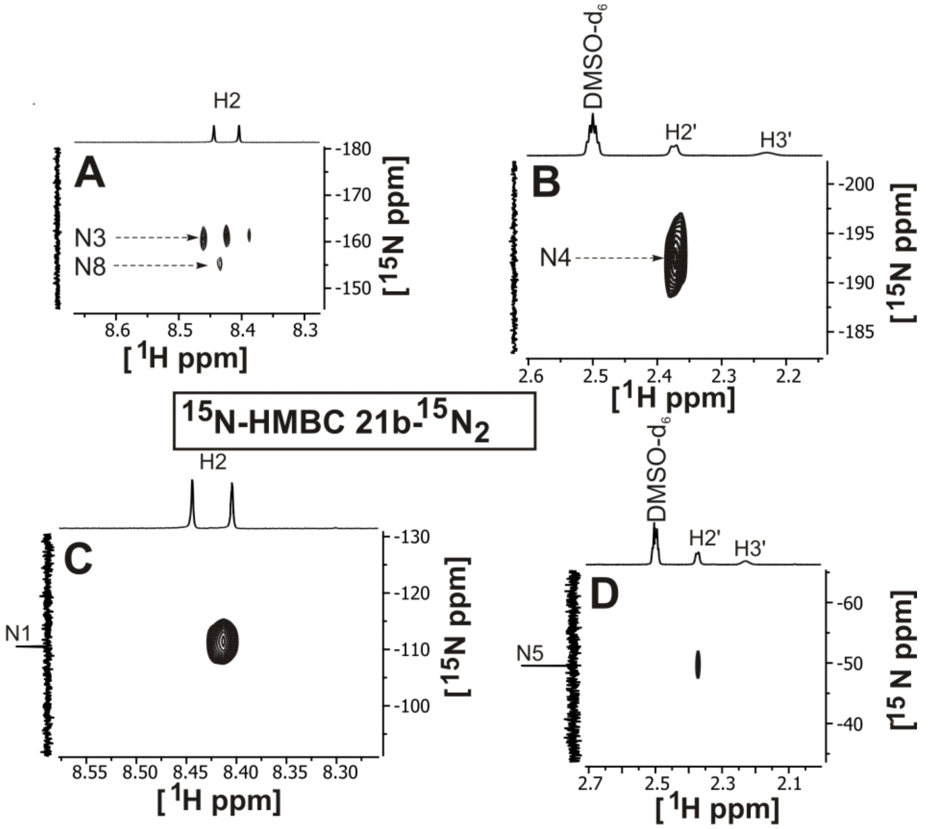

Figure S22: Fragments of the 2D ^{15}N HMBC spectrum of 32 mM **21b-^{15}N$_2$** measured in DMSO-d_6 (400 MHz, 45 °C). The separate panels show the correlation peaks for the nitrogen atoms at natural isotopic abundance (**A** and **B**) and for the ^{15}N-labelled atoms (**C** and **D**).

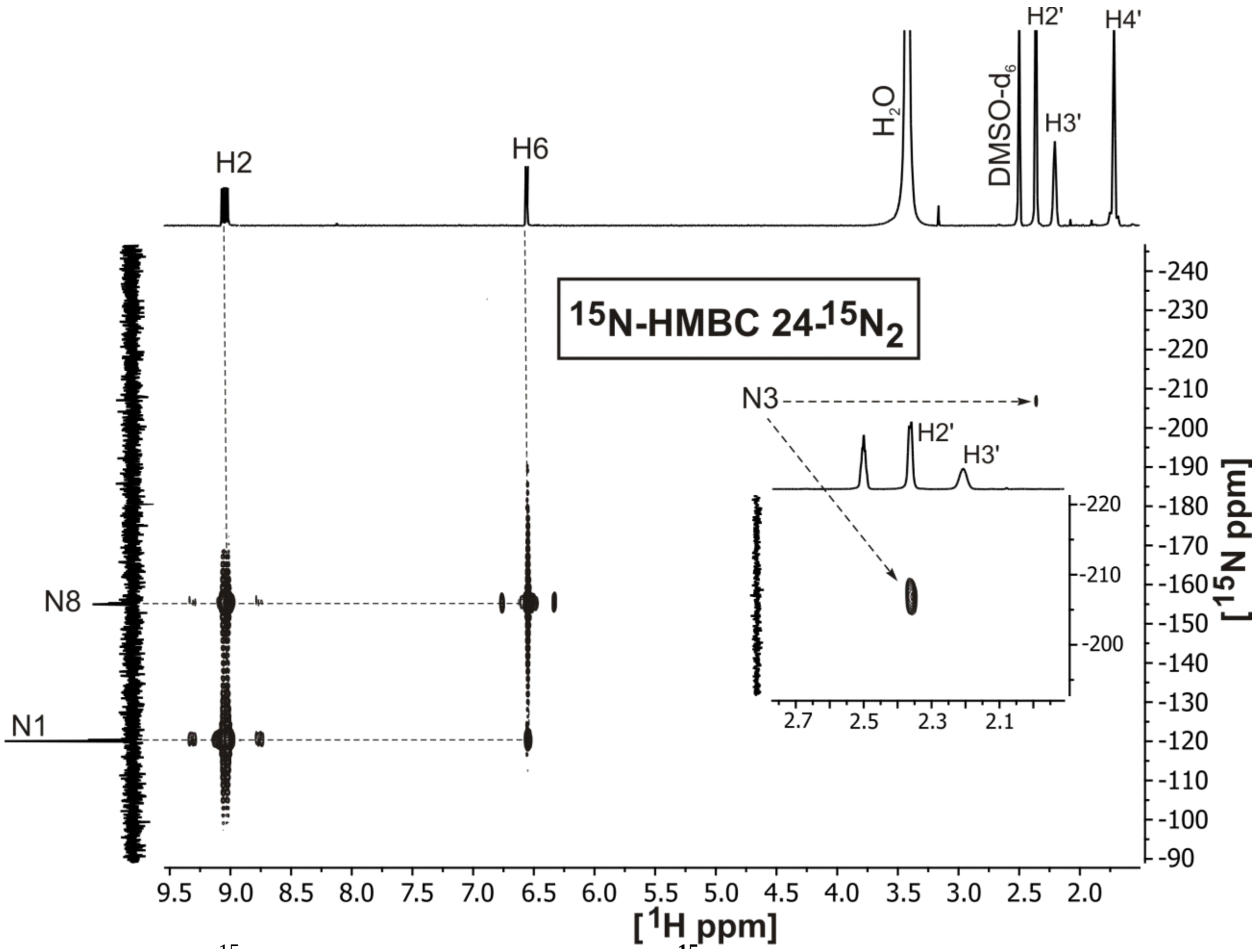

Figure S23: 2D ^{15}N HMBC spectrum of 53 mM **24-^{15}N$_2$** measured in DMSO-d_6 (400 MHz, 45 °C). Inset shows the enlarged part of the spectrum with additional intensity scaling.

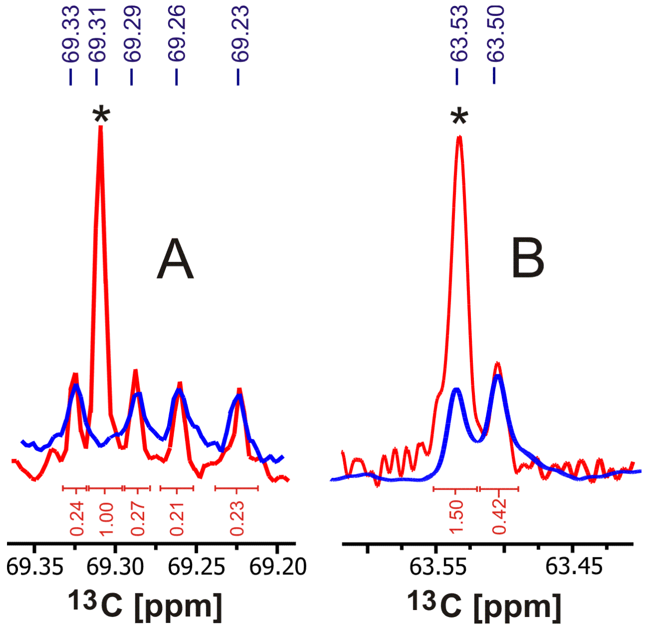

Figure S24: Fragments of the 1D ^{13}C spectra of **15a** (**A**) and **15b** (**B**) (DMSO-d_6, 400 MHz, 45 °C). The spectra of the pure ^{15}N-labelled compounds (^{15}N enrichment of 98%) are shown in blue lines. The spectra of the mixture of ^{15}N-labelled/unlabelled compounds (^{15}N enrichment of 49%) are shown in red lines. Signals of C1' atoms are shown. The resonances of unlabelled compounds are marked by an asterisk (*). The degree of ^{15}N enrichment was determined by

mass spectrometry and confirmed by the ratio of integral intensities of different components in the ^{13}C spectra.

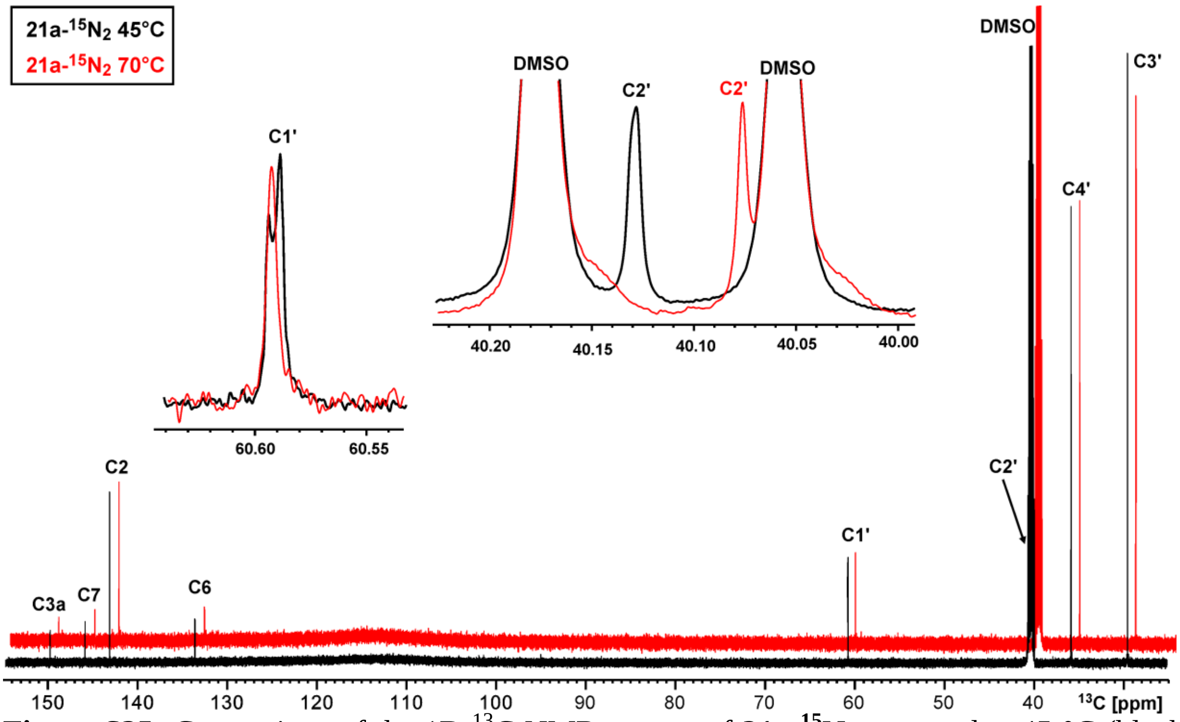

Figure S25: Comparison of the 1D ^{13}C NMR spectra of **21a-^{15}N$_2$**measured at 45 °C (black) and 70 °C (red). The spectra were acquired with broadband ^{1}H decoupling and band-selective ^{15}N1 decoupling using a 700 MHz NMR spectrometer. Insets show the fragments with the C1' and C2' resonances.
